# Nanoscale Chemical Analysis of Heterogeneous Catalysts Using Tip-Enhanced Raman Spectroscopy

**DOI:** 10.1021/acs.chemrev.5c00707

**Published:** 2026-02-04

**Authors:** Naresh Kumar, Li-Qing Zheng, Andrew J. Pollard, Andrew J. Wain, Renato Zenobi

**Affiliations:** † Department of Chemistry and Applied Biosciences, 27219 ETH Zurich, Zurich CH-8093, Switzerland; ‡ State Key Laboratory of Analytical Chemistry for Life Science, School of Chemistry and Chemical Engineering, Nanjing University, Nanjing 210023, People’s Republic of China; § 9917 National Physical Laboratory, Hampton Road, Teddington TW11 0LW, United Kingdom

## Abstract

Heterogeneous catalysts underpin much of the modern chemical industry, yet their rational design for enhanced activity, selectivity, and sustainability remains a formidable challenge due to the intrinsic structural and chemical heterogeneity of catalytic surfaces. Conventional ensemble-averaged characterization techniques often fail to capture the nanoscale complexity that governs catalytic function. Over the past two decades, tip-enhanced Raman spectroscopy (TERS) has emerged as a powerful nanoanalytical technique, offering single-molecule sensitivity and spatial resolution down to the Ångström scale. In this Review, we present TERS as a versatile, nondestructive, and label-free approach for probing heterogeneous catalytic reactions with nanometer-scale chemical specificity in air, liquid, and electrochemical environments. We first introduce the fundamental principles and instrumental implementations that underpin reliable TERS measurements. We then provide a comprehensive and critical assessment of reported *ex situ*, *in situ*, and emerging *operando* TERS studies across a wide range of catalytic systems, highlighting key mechanistic insights uniquely accessible by this technique. Finally, we discuss the technical challenges and methodological requirements for advancing *operando* TERS toward realistic reaction conditions, and outline promising directions for future research. By integrating practical considerations with conceptual advances, this Review aims to serve as a comprehensive guide for researchers seeking to apply TERS to nanoscale chemical analysis in heterogeneous catalysis.

## Graphical abstract

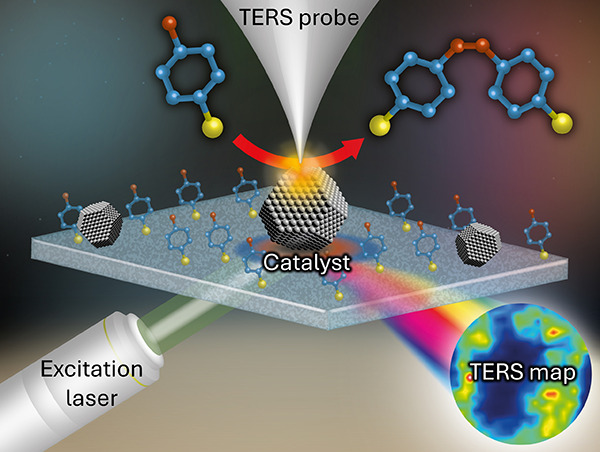

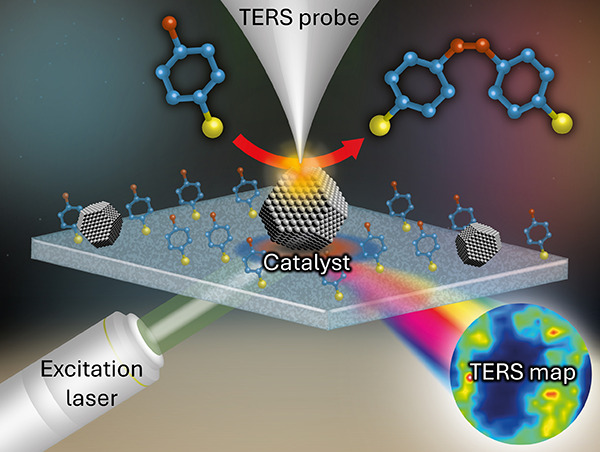

## 1 Introduction

Heterogeneous catalysis plays a pivotal role in driving technological advancements aimed at achieving sustainability, reducing environmental impact, and minimizing reliance on fossil fuels. It is fundamental to the modern chemical industry, contributing to the production of 80–90% of all chemical products^1^ and accounting for nearly 35% of the global gross domestic product.^2,3^ Heterogeneous catalysts, such as supported metal nanoparticles and zeolites, enable efficient, large-scale, and selective chemical transformations that would otherwise be economically unviable or synthetically inaccessible.^4^ Similarly, electrocatalysts are central to the advancement of green energy conversion technologies, including hydrogen fuel cells and electrolyzers.^5^ The ongoing pursuit of greener, more cost-effective, and sustainable catalytic processes is driving the development of novel catalysts based on earth-abundant elements with enhanced activity and selectivity.^6^ The rational design and optimization of catalytic materials is underpinned by a detailed understanding of their operational mechanisms, including the nature of active sites, reaction pathways, and deactivation processes. This necessitates advanced analytical techniques capable of probing catalytic surfaces with molecular specificity and nanometer to subnanometer spatial resolution. Complicating this challenge is the dynamic behavior of catalytic sites, which often vary under different reaction conditions, emphasizing the critical need for *in situ* or *operando* characterization.

While conventional spectroscopic methods, such as Raman,^7^ infrared (IR),^8^ fluorescence,^9^ and UV–vis^10^ spectroscopies, offer molecular insights, they are typically limited by insufficient sensitivity or spatial resolution. Conversely, a range of cutting-edge nanoscale characterization tools, including nanoscale secondary ion mass spectrometry (NanoSIMS),^11^ atom probe tomography (APT),^12^ transmission electron microscopy (TEM),^13^ nanoscale infrared (Nano-IR) spectroscopy,^14^ and nanoscale X-ray fluorescence (Nano-XRF),^15^ can provide high-resolution structural or compositional data. For instance, remarkable progress has been made in applying *in situ* TEM for the direct visualization of electrochemical energy-storage systems including Li–S batteries^16^ and charge/ion accumulation phenomena^17^ in working microcells. However, extending these approaches to truly realistic heterogeneous catalytic reaction conditions (e.g., complex reactive gas mixtures at near-ambient to bar-level pressures, elevated temperatures, and with minimal electron-beam-induced artifacts) remains challenging.

In this context, tip-enhanced Raman spectroscopy (TERS) has emerged as a powerful technique for nanoscale molecular characterization of heterogeneous and electrochemical catalytic systems. Since its first demonstration in 2000,^18–21^ TERS has evolved into a versatile nanoanalytical tool by integrating Raman spectroscopy with scanning probe microscopy (SPM). In TERS, ultrahigh molecular sensitivity and nanoscale spatial resolution originate from the strongly confined and enhanced electromagnetic near field generated at the apex of a plasmonic tip through localized surface plasmon resonance (LSPR). The SPM platform provides precise control over the tip–sample distance and enables raster scanning of the plasmonic tip across the surface, thereby facilitating spatially resolved mapping of chemically specific Raman signals with nanometer-scale resolution. TERS has been successfully applied across diverse fields,^22,23^ including one-dimensional (1D) materials such as carbon nanotubes,^24–27^ two-dimensional (2D) materials such as single-layer graphene,^28–36^ transition metal dichalcogenides,^37–45^ and 2D polymers;^46,47^ organic photovoltaics;^48,49^ biological systems including cells^50–52^ and lipid membranes;^53,54^ polymer blends;^55–57^ photoisomerization dynamics;^58–61^ self-assembled monolayers (SAMs);^62–65^ solid–liquid interfaces;^66^ heterogeneous catalysis and electrocatalysis.^67–72^

The key advantage of TERS is its ability to provide correlative information about catalyst surface morphology and molecular fingerprint information on the catalytic transformations with ultrahigh sensitivity and nanoscale spatial resolution. This allows direct correlation of surface topography features such as steps, edges and terraces with corresponding catalytic activity providing valuable information for mechanistic understanding of catalytic transformation and optimization of catalytic efficiency. TERS is also compatible with both ambient and liquid environments, positioning it well for *operando* studies of catalytic reactions.^66,73–76^ Consequently, the number of TERS studies focused on catalytic systems have been rising steadily over the past decade.

In this review, we provide a comprehensive account of TERS-based investigations of catalytic systems reported to date. We identify the catalytic materials and reaction systems where TERS characterization has proven most impactful. The article is structured into two main sections. In the first section, we present the fundamental operating principles of TERS, including instrumentation details, experimental considerations, and common challenges alongside practical solutions. In the second, we offer an in-depth account of TERS studies on various catalytic systems, focusing on the insights gained and their implications. We conclude with a critical discussion on future directions for TERS development and provide practical guidelines for its effective application in catalytic research.

## 2 TERS Fundamentals

### 2.1 TERS Principle

Raman spectroscopy is a widely used analytical technique for molecular identification based on characteristic vibrational fingerprints. However, the inherently weak Raman scattering cross-section severely limits its sensitivity, making the detection of low surface coverages such as SAMs of organic molecules challenging using conventional far-field Raman spectroscopy. In addition, the spatial resolution of confocal Raman microscopy is restricted by the optical diffraction limit to approximately 200–300 nm under visible excitation. Both these limitations can be overcome by exploiting the phenomenon of LSPR. Upon irradiation of metallic nanostructures, incident electromagnetic radiation induces collective oscillations of conduction electrons called localized surface plasmons (LSPs).^77^ When the excitation frequency matches the natural resonance frequency of these oscillations, LSPR is established, resulting in a strong enhancement of the local electromagnetic (EM) field at the metal surface. In TERS, LSPR is excited by positioning a sharp metallic SPM tip within the focal volume of the excitation laser (Figure 1a).^78^ The nanoscale curvature of the tip apex supports a highly confined plasmonic mode which, in combination with the lightning rod effect (LRE),^79^ leads to high EM field enhancement and spatial confinement at the probe apex. Numerical simulations demonstrate that this enhanced EM field is localized to a nanometric volume beneath the tip apex (Figure 1b).^22^ The resulting intense near-field amplifies Raman scattering from molecules located beneath the probe apex by several orders of magnitude, enabling chemical analysis with nanoscale spatial resolution and ultrahigh sensitivity. This plasmonically confined near-field interaction constitutes the fundamental physical basis of TERS and underlies its application to nanoscale chemical analysis of heterogeneous catalytic systems.

**Figure 1:**
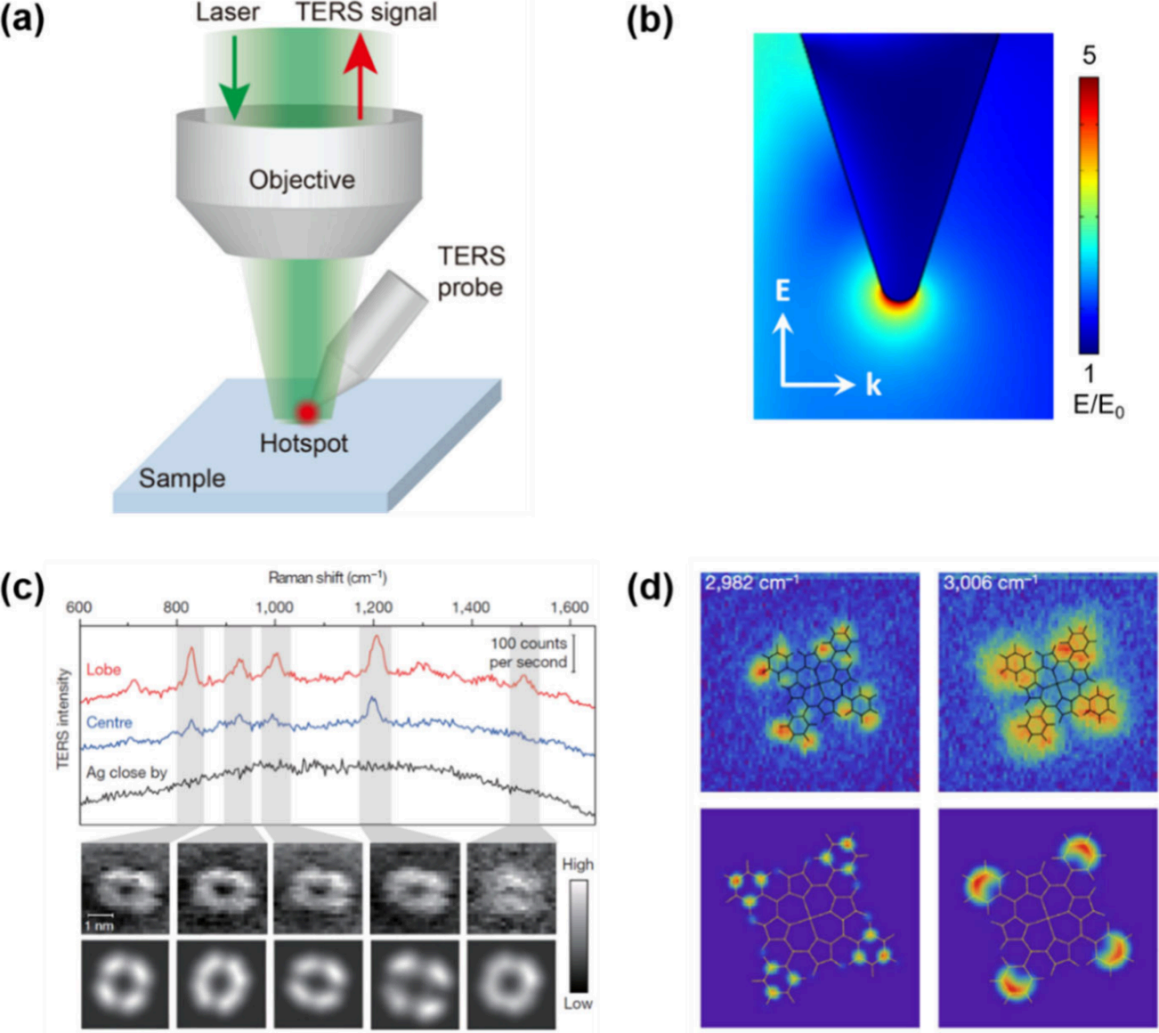
(a) Schematic illustration of the working principle of TERS. (b) Simulated spatial distribution of the electric field enhancement (*E*/*E*_0_, where *E* and *E*_0_ denote the enhanced and incident electric field amplitudes, respectively) at the apex of a Ag TERS probe irradiated with a 532 nm excitation laser, with the electric field polarized parallel to the probe axis. The radius of the probe apex is 15 nm. (c) Single-molecule TERS imaging of a *meso*-tetrakis­(3,5-di-*tert*-butylphenyl)­porphyrin (H_2_TBPP) molecule adsorbed on an Ag(111) surface.^94^ (top) Representative TERS spectra acquired at the molecular lobe (red) and center (blue) of a flat-lying H_2_TBPP molecule, together with a spectrum measured on the bare Ag surface approximately 1 nm away from the molecule (black). Integration time: 3 s. (middle) TERS images (23 × 23 pixels) mapping the intensity of selected Raman bands of a single H_2_TBPP molecule. Integration time: 0.3 s; step size: 0.16 nm. (bottom) Corresponding theoretical simulations of the TERS images. Adapted with permission from ref 94. Copyright 2013 Springer Nature. (d) Single-molecule TERS imaging of Co­(II)–tetraphenylporphyrin (CoTPP) adsorbed on a Cu(100) surface.^95^ (top) TERS images (64 × 64 pixels) of the 2982 and 3006 cm^–1^ vibrational modes with overlaid molecular frameworks. Integration time: 2 s; step size: 0.29 nm. (bottom) Corresponding theoretical simulations of the vibrational mode distributions. Adapted with permission from ref 95. Copyright 2019 Springer Nature.

It should be noted that while LSPR is a resonant phenomenon arising from the collective oscillation of conduction electrons at a metal nanostructure at specific excitation frequencies, LRE is a nonresonant field enhancement mechanism associated with geometric charge concentration at sharp metallic features.^80^ Additionally, TERS can also exhibit a chemical enhancement (CE) contribution for certain analytes that interact strongly with the plasmonic tip or substrate.^81,82^ The CE effect originates from molecule–metal electronic interactions that transiently modify the molecular polarizability and Raman cross-section via charge transfer (CT) processes between the adsorbed molecule and the metallic nanostructure.^83^ In the CE mechanism, the formation of a metal–molecule complex or CT resonance can lead to additional enhancement when the incident photon energy is resonant with an interfacial CT transition, which depends on the relative energies of the molecule’s frontier orbitals (HOMO/LUMO) and the Fermi level of the plasmonic metal.^84^ However, since this effect requires intimate electronic coupling and favorable energy alignment, it is a short-range, molecule-specific contribution that is typically much smaller and not universally present compared to the plasmonic EM enhancement.

The magnitude and spectral position of the near-field enhancement in TERS depend sensitively on the tip material, geometry, and measurement configuration. In practice, Ag tips typically provide stronger enhancement at shorter visible wavelengths, with resonances often better matched to blue–green excitation, whereas Au tips exhibit greater chemical stability and resonances commonly better matched to red/near-IR excitation.^85–87^ The LSPR response can be tuned by the tip geometry including the apex radius, cone angle, and overall taper, as established through EM modeling and experimental investigations.^87–90^ Engineered tip designs, such as metal-coated dielectric probes provide additional routes to tune the plasmon resonance frequency and optimize enhancement at a desired excitation wavelength.^85^ In addition, the tip–sample coupling strongly modifies the resonance: “gap-mode” TERS (metal tip over a metallic substrate) can produce substantially larger field confinement and enhancement than non-gap configuration (metal tip over a dielectric substrate) due to plasmon hybridization across the nanogap,^91^ while the local dielectric environment and substrate morphology can shift the effective resonance and the EM enhancement.^92,93^

These enhancement effects allow TERS to overcome the diffraction limit associated with conventional Raman spectroscopy or surface-enhanced Raman spectroscopy (SERS) and provide nondestructive, label-free nanoscale molecular imaging under ambient conditions. Remarkably, the latest advances in TERS have even demonstrated imaging of vibrational modes within individual molecules, resolving single-bonds with Ångström-scale spatial resolution, as illustrated in Figure 1c,d.^94–96^ However, such ultrahigh-resolution imaging relies on the stable atomic-scale confinement of optical fields at the atomic protrusions at the apex of metallic TERS tips, which is only achievable under ultrahigh vacuum (UHV) and cryogenic conditions.^97,98^

### 2.2 Experimental Configurations

#### 2.2.1 Top, Bottom, Side, and Parabolic Mirror Illumination

The optical excitation geometry in a TERS setup can be broadly categorized into four configurations: top, bottom, side, and parabolic mirror illumination, as schematically illustrated in Figure 2a–d, respectively.^99^ Top, side, and parabolic mirror illumination geometries are commonly grouped under reflection-mode TERS, whereas bottom illumination is referred to as transmission-mode or back-reflection TERS. In the top-illumination configuration (Figure 2a), the excitation laser is focused from above onto the apex of a tilted scanning tunnelling microscopy (STM) probe or a metallic “nose-type” atomic force microscopy (AFM) probe.^100^ This geometry enables efficient coupling of the incident light to the probe apex and is widely used in both STM- and AFM-based TERS systems. In contrast, bottom illumination (Figure 2b) involves focusing the laser through a transparent substrate onto the sample using a high numerical aperture (NA, typically >1) objective lens.^23^ While this transmission-mode geometry is relatively straightforward to implement and provides efficient light collection, it requires both the sample and substrate to be optically transparent, thereby limiting the range of materials that can be investigated.

**Figure 2:**
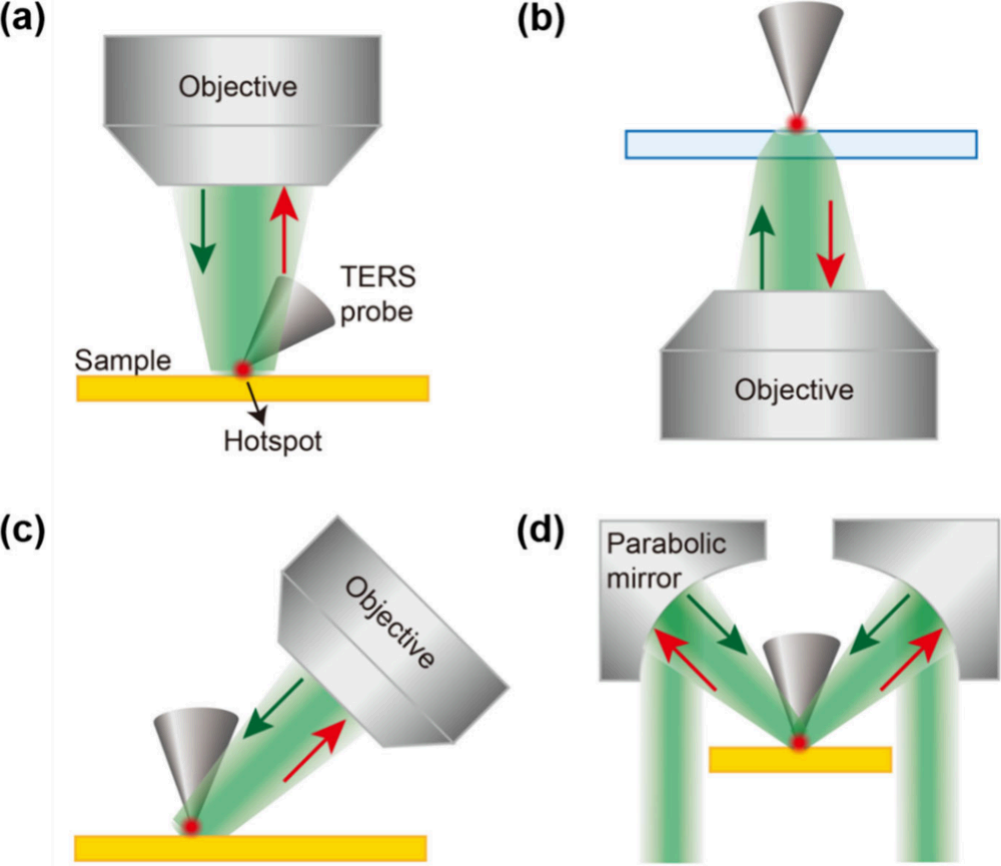
Schematic representations of the principal optical excitation geometries used in TERS: (a) top illumination, (b) bottom illumination (transmission or back-reflection mode), (c) side illumination, and (d) parabolic mirror-based illumination.

In side illumination (Figure 2c), a linearly polarized laser beam is focused onto the probe apex from the side using a long working distance objective lens.^32^ By aligning the laser polarization parallel to the probe axis, strong EM field enhancement can be achieved even with moderate NA optics. As a result, side- and top-illumination reflection-mode configurations are particularly well suited for nanoscale chemical analysis of opaque samples or samples supported on nontransparent substrates.

Parabolic mirror illumination (Figure 2d) represents a less common reflection-mode configuration, in which the excitation laser is focused onto the TERS probe apex using a parabolic mirror.^95,101^ Despite its relatively limited adoption, this geometry offers several advantages, including the absence of chromatic aberration, a high effective NA approaching unity, and tight focusing of the excitation beam at the probe apex, making it an attractive option for high-resolution TERS measurements. Notably, in contrast to bottom-illumination geometry (Figure 2b), the TERS tip in top- , side- and parabolic mirror illumination configurations (Figure 2a,c,d) can partially obstruct the incident laser beam. Nevertheless, LSPR can still be efficiently excited at the tip apex in these configurations. Moreover, unlike bottom illumination, where the TERS signal is attenuated upon transmission through the glass substrate, top- and side-illumination geometries allow free-space collection of Raman-scattered light originating from the plasmonic near field, thereby enabling TERS imaging with high sensitivity and nanoscale spatial resolution. A summary of the advantages and limitations of different illumination geometries in TERS is presented in Table 1.

**Table 1:** Advantages and Limitations of Different TERS Illumination Geometries

illumination mode	advantages	limitations
top (reflection mode)	applicable to opaque samples; free-space collection of Raman scattered light avoids substrate-induced attenuation; efficient excitation of LSPR at the tip apex	partial obstruction of the incident laser by the tip; requires specific probe geometries and careful alignment
bottom (transmission/back-reflection mode)	relatively simple optical implementation; high-NA focusing enables strong excitation	requires transparent samples, limiting applicability; TERS signal suffers attenuation upon transmission through the substrate
side (reflection mode)	strong electromagnetic enhancement achievable by aligning polarization with tip axis even at moderate NA; suitable for opaque samples; free-space signal collection enables high sensitivity	partial blocking of the incident beam by the tip; requires precise polarization control, side optical access and careful alignment
parabolic mirror (reflection mode)	no chromatic aberration; tight laser focusing with high NA; suitable for opaque samples; efficient LSPR excitation	less common and experimentally more complex; partial obstruction of the incident beam by the tip; specialized setup required

#### 2.2.2 SPM Feedback

From the perspective of SPM feedback, TERS instrumentation can be grouped into three main classes: (1) contact- and tapping-mode AFM,^102^ (2) shear-force AFM,^102^ and (3) STM.^103^ These configurations are schematically depicted in Figure 3. Because AFM can probe both conductive and insulating samples, it forms the basis of many bespoke and commercial TERS platforms. In AFM operation, a feedback loop maintains a defined tip–sample interaction force. Variations in this force deflect the cantilever and are detected optically, typically by reflecting a laser beam from the back of the cantilever onto a quadrant photodiode detector (Figure 3a). Ag-coated AFM–TERS probes commonly deliver spatial resolutions better than 20 nm.^69^ Beyond contact mode, reliable TERS measurements have also been demonstrated in tapping mode,^104,105^ where the signal depends sensitively on the oscillation amplitude; notably, lower tapping amplitudes generally yield higher TERS intensities.^106^ In shear-force AFM, a metallic wire probe is attached to a prong of a quartz tuning fork (Figure 3b). Here, damping of the tuning-fork oscillation provides the feedback signal, enabling particularly stable and fine control of the tip–sample separation, often superior to that achievable in conventional contact or tapping modes for TERS measurements.^107^

**Figure 3:**
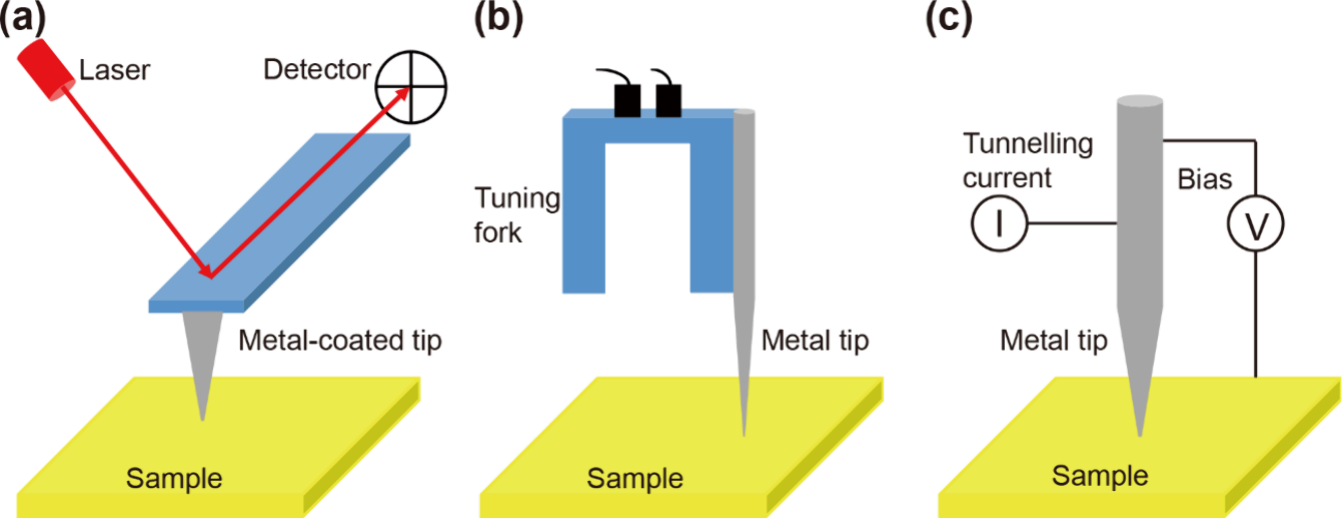
Schematic representations of the SPM feedback mechanisms employed in TERS: (a) contact- and tapping-mode AFM, (b) shear-force AFM, and (c) STM.

In STM–TERS, the probe–sample distance is regulated by a feedback loop based on the tunnelling current between an etched metallic tip and the sample (Figure 3c).^71^ The requirement for electrical conductivity means that most STM–TERS studies are performed on opaque metallic substrates and therefore commonly employ reflection-mode optical geometries, although transmission-mode STM–TERS using metal-coated transparent substrates has also been reported.^21^ As a result, the range of compatible samples is generally narrower than for AFM–TERS. The major advantage, however, is spatial resolution: atomically sharp STM tips have enabled TERS imaging with submolecular resolution reaching 2–5 Å ^94,95^ under cryogenic UHV conditions. UHV–STM–TERS therefore provides a unique pathway toward resolving the nature and evolution of chemical bonds at the atomic scale in surface-catalyzed reactions. For example, Jiang and co-workers employed UHV–STM–TERS to chemically identify individual adatoms with single-bond sensitivity during the oxidation of borophene on Ag(111) at 78 K with a spatial resolution of 4.8 Å.^108^ In a separate study, the same group used UHV–STM–TERS to probe N-heterocyclic carbenes (NHCs) on borophene at the single-molecule level, also at 78 K and with subnanometer resolution.^109^ Their measurements revealed two distinct interfacial states between individual NHCs and borophene, corresponding to covalent (boron–carbon) bonding and van-der-Waals-type interactions. These works highlight the still untapped potential of UHV–STM–TERS for mechanistic investigations of surface reactions with atomic-scale precision.

### 2.3 Signal Enhancement in TERS

#### 2.3.1 Laser Polarization

In TERS, the polarization state of the excitation laser is a key determinant of how efficiently LSPR is excited at the probe apex. Efficient LSPR excitation is achieved when the incident electric field is aligned parallel to the probe axis (p-polarization),^94^ whereas a field oriented perpendicular to the probe axis (s-polarization) typically produces little to no appreciable EM enhancement. Consequently, in reflection-mode geometries employing top or side illumination (Figure 2a,b), linearly polarized excitation with the polarization aligned along the probe axis is generally preferred to maximize near-field enhancement. In transmission-mode bottom illumination (Figure 2c), however, the situation is more nuanced because tight focusing with a short working distance, high-NA objective redistributes the vectorial field components at the focus. Under these conditions, radially polarized excitation can generate a focal field with a dominant Z-component (*Z*-axis normal to the sample), which couples more efficiently to the tip axis and yields stronger EM enhancement.^110,111^ Indeed, for bottom illumination, the EM enhancement obtained with radial polarization can exceed that achieved with linear polarization by more than a factor of 4.^112,113^

#### 2.3.2 Enhancement Factor

In the near-field, the plasmonically enhanced region localized at the apex of a TERS probe, the Raman scattering intensity scales with the fourth power of the local EM field enhancement (∝ |E|^4^).^114,115^ As a result, even modest increases in the local EM field amplitude can lead to pronounced amplification of Raman signals from molecules residing in the near-field, relative to the far-field signal generated in the focal volume of the excitation laser in the absence of the TERS probe. The enhancement factor (*EF*) of the TERS signal is defined as^116^

1 $$EF=\left(\frac{I_{\mathrm{Tip‐in}}}{I_{\mathrm{Tip‐out}}}-1\right)\frac{V_{\mathrm{FF}}}{V_{\mathrm{NF}}}$$

where *I*_Tip‑in_ and *I*_Tip‑out_ denote the Raman intensities measured with the TERS probe engaged with (near-field + far-field contributions) and retracted from (far-field contribution only) the sample, respectively. The subtraction of unity removes the far-field background, isolating the net near-field-induced enhancement. The terms *V*_NF_ and *V*_FF_ represent the effective sample volumes contributing to the near-field and far-field Raman signals, respectively. In general, *V*_NF_ is defined by the spatial extent of the highly confined plasmonic near field at the tip apex, typically on the order of a few nanometers laterally and vertically, whereas *V*_FF_ corresponds to the much larger diffraction-limited focal volume of the incident laser. For bulk samples, the electromagnetic penetration depths of both near-field and far-field components depend strongly on the dielectric properties of the material,^117^ making an accurate determination of *V*_NF_ and *V*_FF_ nontrivial and rendering EF estimation highly model-dependent. However, for thin-film systems, such as 2D materials or SAMs, the situation is significantly simplified. In these cases, the effective interaction volumes reduce to surface areas because the sample thickness is much smaller than both the near-field decay length and the optical penetration depth. Consequently, *V*_NF_ and *V*_FF_ can be approximated by the respective near-field and far-field sampling areas, enabling a more reliable and reproducible estimation of the TERS enhancement factor.

However, for TERS imaging, a more practically relevant metric is the contrast, defined as^116^

2 $$\mathrm{Contrast}=\frac{I_{\mathrm{Tip‐in}}}{I_{\mathrm{Tip‐out}}}-1$$

In practice, contrast serves as a more convenient and reliable indicator of TERS probe performance than absolute enhancement factors, as it is straightforward to determine experimentally and enables direct comparison of the far-field and near-field Raman signals.

#### 2.3.3 Spatial Resolution

Another commonly reported metric in the TERS literature is spatial resolution, which has been estimated using several different approaches. A commonly used method is to determine the full width at half-maximum of the TERS signal across an isolated nanoscale feature in a line profile or 2D image. While straightforward, this approach is highly sensitive to the intrinsic size and shape of the underlying sample feature and must therefore be applied cautiously.^95^ An alternative and frequently adopted approach is based on imaging a sharp chemical or structural step edge, where the spatial resolution is extracted using a 10–90% intensity criterion across the step. This method reduces ambiguity associated with feature size but requires well-defined, abrupt boundaries and sufficient signal-to-noise ratio.^118^ In some reports, spatial resolution has been inferred directly from the pixel or step size used during hyperspectral imaging.^119^ While this may be justified in cases where the TERS signal appears exclusively within a single pixel and is reproducible upon repeated scans, this approach provides, at best, an upper bound on the achievable resolution and must be interpreted in light of sampling considerations such as the Nyquist criterion.^120,121^ For thick samples, where the far-field Raman contribution can be substantial, accurate determination of TERS spatial resolution is particularly challenging because the measured signal is a convolution of near-field and far-field components. Reliable estimation therefore requires explicit separation of these contributions, which can be achieved by identifying a region of genuine spectral contrast associated with a realistic surface feature and modeling the intensity profile as the sum of near-field and far-field Gaussian components.^23^ Deconvolution of these components yields a more physically meaningful estimate of the near-field spatial resolution. Overall, the reported spatial resolution in TERS depends strongly on experimental conditions (e.g., drift, tip stability, enhancement factor, sampling density etc.), and no single metric is universally applicable.^97^ Best practice therefore requires clearly stating the method used, justifying its validity for the specific sample system, and reporting relevant experimental parameters.

### 2.4 TERS Probes

#### 2.4.1 Coated Probes

TERS probes lie at the heart of TERS experiments, since their plasmonic activity critically determines experimental performance. Accordingly, careful preparation, protection, and storage of TERS probes are essential for reliable and reproducible measurements. Due to their strong LSPR in the visible–near-IR and comparatively low damping losses, Ag and Au are the most widely used materials for TERS probes, enabling excitation of intense and highly confined near fields at the tip apex.^122^ Ag tips often deliver particularly large enhancements because Ag supports sharper (less damped) plasmon resonances in the visible, whereas Au tips typically offer superior chemical stability (resistance to oxidation/sulfidation) and robust performance under ambient/*operando* conditions. A detailed discussion of the factors governing the TERS enhancement is provided in section 2.1.

The earliest TERS experiments were reported two and a half decades ago by Zenobi and co-workers using Ag-coated Si AFM probes.^18^ Since then, Ag- and Au-coated probes produced by high-vacuum (HV) thermal evaporation (typically <10^–6^ mbar) have been widely adopted for TERS measurements (Figure 4a). Although commercially available metal-coated AFM–TERS probes have recently emerged, and several groups have demonstrated alternative fabrication approaches, such as direct attachment of plasmonically active Ag nanoparticles^123^ or Ag nanowires^124^ to AFM tips, in-house metallization of standard AFM probes remains a common and cost-effective strategy. A longstanding challenge associated with metal coating is the typically low yield of probes exhibiting strong and reproducible signal enhancement. This limitation arises from the stochastic nucleation and growth of metal nanostructures near the probe apex during deposition, as illustrated by the SEM image of a representative Ag-coated TERS probe in Figure 4b. Significant progress toward overcoming this issue has been made by modifying the optical properties of the probe surface prior to metallization. In particular, reducing the effective refractive index at the probe apex has been shown to markedly improve the plasmon resonance characteristics and reproducibility of TERS probes. For example, Yeo et al. reported improved yield of probes with high *EF* by coating AFM cantilevers with thin layers of SiO_2_, SiO_*x*_, or AlF_3_ prior to Ag deposition.^125^ Similarly, Hayazawa et al. achieved nearly 100% yield of plasmonically active TERS probes by thermally oxidizing Si AFM cantilevers to form approximately 300 nm thick SiO_2_ layer before Ag coating.^126^

**Figure 4:**
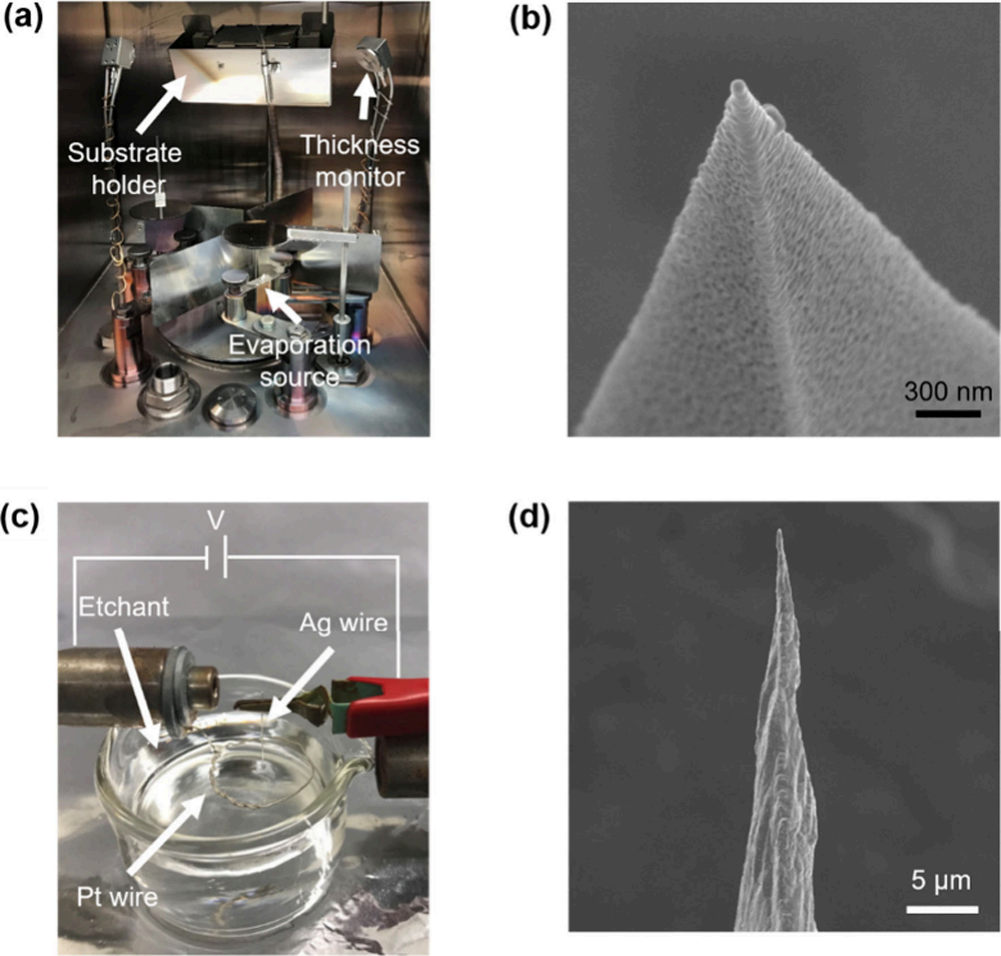
(a) Photograph of a vacuum evaporation chamber used for the fabrication of TERS probes by thermal deposition of Ag or Au onto commercial AFM tips. (b) SEM image of a representative Ag-coated TERS probe. (c) Photograph of an experimental setup used for the preparation of electrochemically etched Ag or Au TERS probes. (d) SEM image of a representative electrochemically etched Ag TERS probe.

Beyond conventional vacuum deposition, electrochemical deposition-based fabrication of metal-coated AFM–TERS probes has emerged as an attractive alternative, offering improved control over coating morphology and reproducibility. Using pulsed or potentiostatic electrodeposition, uniform and conformal noble-metal coatings can be deposited directly onto AFM probes, yielding enhanced plasmonic activity, improved probe-to-probe consistency, and, in some cases, improved robustness in demanding environments compared with stochastic thermal evaporation.^127,128^ These advances highlight the critical role of probe engineering in achieving reliable and high-performance TERS measurements.

#### 2.4.2 Etched Probes

Electrochemically etched Ag and Au metal probes are predominantly used in STM–TERS and can be reproducibly fabricated using drop-off etching protocols.^129–138^ This approach yields sharp metallic apices capable of supporting strong plasmonic confinement. Etched Ag probes, typically produced from Ag wires, often exhibit apex diameters on the order of tens of nanometers (Figure 4d) and show high reproducibility, with up to ∼90% of freshly prepared probes displaying strong plasmonic enhancement in TERS measurements.^139^ Etched Au probes can be prepared using analogous electrochemical etching strategies.^140^ Compared to Ag, Au probes offer superior chemical stability under ambient conditions and can retain plasmonic activity over extended periods. However, for probes of comparable geometry, Au generally provides lower signal enhancement than Ag. Accordingly, the choice between etched Ag and Au probes reflects a trade-off between enhancement efficiency and environmental stability, and should be guided by the specific experimental requirements of the TERS study.

Additionally, groove-structured tips have been developed to further enhance plasmonic confinement and improve excitation efficiency.^141^ By introducing engineered grooves near the probe apex (e.g., via focused ion beam milling) of electrochemically etched probes, tailored plasmonic modes can be supported that improve LSPR - excitation wavelength matching and field localization relative to smooth coated tips, thereby boosting enhancement and reproducibility.

#### 2.4.3 Plasmon-Engineered Probes

Recent advances have also introduced plasmon-tunable pyramidal tip platforms (PTTP), which enable deterministic tuning of the tip plasmon resonance through controlled geometry of a pyramidal monopole antenna.^142^ This approach provides improved spectral matching between the excitation wavelength and the tip resonance, offering a more reproducible and tunable alternative to conventional etched-wire tips for maximizing near-field enhancement. Furthermore, nanowire-assisted selective-coupling probe designs have been demonstrated to achieve highly efficient and controllable plasmonic excitation.^143^ In this strategy, a nanowire-based coupler enables selective launching and nanofocusing of optical energy into the tip–sample junction, which can significantly suppress far-field background and enhance field confinement, thereby improving sensitivity and spatial resolution.

#### 2.4.4 Enhancing Lifetime of TERS Probes

Despite improvements in probe yield, the plasmonic lifetime of TERS probes, particularly coated or electrochemically etched Ag tips, remains limited under ambient conditions, posing a major challenge. Kumar et al. showed that Ag-coated TERS probes undergo rapid degradation in the ambient environment, losing their plasmonic enhancement in under 3 h due to surface Ag oxidation.^144^ Similarly, Opilik et al. reported that the *EF* of electrochemically etched Ag TERS probes dramatically decreases within 48 h storage in ambient environment due to Ag oxidation.^139^ Degradation of the Ag probes led to a broad Raman band in the 180–300 cm^–1^ region, assigned to Ag_2_S and/or Ag_2_O formation, accompanied by a sharp band at 960 cm^–1^ attributed to sulfate or sulphite ions.^145,146^ Based on their findings, these studies recommended several strategies to extend the lifetime of Ag probes, including storing them in an oxygen- and moisture-free (inert) environment, protecting the surface with a thin dielectric coating, or performing TERS measurements under inert atmospheric conditions.

Opilik et al. introduced an approach in which Ag TERS probes were coated with 2–4 nm silica layer, resulting in a substantial improvement in probe stability.^147^ The silica coating was formed via surface functionalization followed by controlled silicate condensation, producing a uniform protective shell around the probe apex. Despite the presence of this dielectric layer, the probes retained appreciable TERS enhancement and exhibited remarkably stable performance over extended periods, with reproducible signal enhancement maintained for up to 20 days.^148^ Subsequent studies further demonstrated that silica-coated probes remain stable under laser irradiation over a wide range of excitation intensities, indicating effective suppression of laser-induced nanostructure reshaping at the probe apex.^147^ However, the impact of silica coatings on long-term STM performance needs to be further explored.

Alternative dielectric protection strategies have also been reported. Barrios et al. demonstrated that deposition of ∼3 nm alumina layer by thermal evaporation can effectively stabilize Ag-coated TERS probes, yielding nearly constant plasmonic enhancement for more than 40 days.^149^ Kumar et al. reported the use of an ultrathin zirconia coating to further extend probe lifetime.^69^ In this case, the zirconia layer acted as a dielectric spacer, resulting in an approximately 50% reduction in TERS contrast relative to freshly prepared unprotected probes, due to attenuation of the plasmonic near field. Nevertheless, the plasmonic stability was dramatically enhanced: after 140 days of ambient exposure, the TERS contrast decreased by only ∼43%, confirming sustained plasmonic activity. These studies highlight a fundamental trade-off between near-field enhancement and long-term stability in protected TERS probes. While ultrathin dielectric coatings inevitably attenuate the local EM field, they offer a powerful route toward reliable, long-lived probes suitable for extended and reproducible TERS measurements, particularly under ambient and *operando* conditions.

Several effective strategies have been established to preserve plasmonic activity and enable recycling and reuse of electrochemically etched Ag probes for STM–TERS under UHV, cryogenic conditions. For example, Taber et al. demonstrated *in situ* preparation and validation protocols, including field-directed sputter sharpening and electroluminescence-based junction plasmon characterization, which remove adventitious carbon, optimize tip geometry, and ensure robust, reproducible plasmonic resonances over repeated use in STM–TERS experiments.^150^ Complementarily, Mahapatra et al. demonstrated an Ar^+^ sputtering strategy in UHV that efficiently removes etching residues and molecular contaminants from Ag tips without degrading their morphology or EF.^151^ They further showed that such cleaned probes retain strong TERS activity for more than two months and can be recycled across different molecular systems and excitation wavelengths. These studies provide practical routes to significantly enhance the functional lifetime, reproducibility, and recyclability of Ag STM–TERS probes operated under UHV, cryogenic conditions.

### 2.5 Performing TERS in Liquids

For *in situ* studies of many heterogeneous catalytic reactions and electrocatalytic processes, TERS measurements must be performed in liquid environments. In liquids, the primary limitation of conventional Au/Ag STM–TERS probes arises from interference by faradaic currents rather than short-circuiting. This issue can be mitigated by partially insulating the probe shaft while leaving the apex exposed, as demonstrated in the first EC–TERS implementation by Ren and co-workers.^152^ In AFM–TERS, probe stability in liquids presents an additional challenge, as metal coatings may delaminate upon immersion. Early approaches to improve adhesion employed SiO_x_ interlayers^73^ or TiN coatings protected by alumina,^153^ which enhanced short-term stability but still suffered degradation after prolonged exposure. A significant advance was achieved using a multilayer metal-coating strategy developed by Kumar et al.,^66^ in which sequential Cr/Au/Ag deposition improved coating uniformity and robustness in aqueous environments. Using such probes, nanoscale TERS imaging in water was demonstrated with spatial resolution of 26 nm, comparable to that achieved in air.

When applying TERS to catalytic reactions in liquids, the chemical reactivity of the metallic probe itself becomes critical, particularly for reactions catalyzed by Ag or Au. To prevent probe-induced perturbation, the plasmonic metal surface must be chemically isolated from the reaction environment with minimal loss of *EF*. Ultrathin dielectric coatings provide an effective solution. For example, alumina-protected Ag-coated AFM–TERS probes enabled chemically inert operation without substantial loss of enhancement, allowing nanoscale imaging of photocatalytic reactions with 20 nm resolution in ambient conditions.^67^ A limitation of silica- and alumina-protected probes is their restricted stability across the full pH range.^154^ Zirconia-coated probes reported by Kumar et al.^69^ (section 2.4.3) address this issue by combining high chemical stability over the entire pH range^154^ with favorable catalytic support properties.^155,156^ These probes enabled enhanced chemical inertness, extended lifetime, and stable TERS operation in aqueous environments, including the first demonstration of nanoscale chemical imaging of photocatalytic reactions in liquids.

For STM–TERS in aqueous or electrochemical environments, insulation of the probe shaft with thin nonconducting layers is required to suppress faradaic and capacitive currents, although such coating is not necessary in nonpolar organic solvents.^74^ To electrically insulate STM probes, several chemical coating protocols have been developed. These include the use of polymeric and insulating materials such as polyethylene,^152^ Zaponlack varnish,^157^ paraffin wax,^158^ and commercial nail polish formulations.^159,160^ These coatings effectively passivate the probe shaft while preserving an exposed apex, thereby suppressing unwanted Faradaic currents and enabling stable tunnelling and high-quality TERS measurements in liquids, with reported *EF* values on the order of 10^5^.^157^ Similarly, EC–TERS measurements have been enabled using polyethylene-coated Au probes to suppress leakage currents and achieve reliable *operando* measurements under potential control.^152^ This approach has allowed acquisition of potential-dependent EC–TERS spectra from molecular SAMs on Au(111), demonstrating the capability of EC–TERS to directly correlate molecular structure and electrochemical behavior at the nanoscale.

To maximize optical collection efficiency and minimize aberrations in EC–TERS, water-immersion objectives with high NA are often employed. This approach reduces optical distortion arising from refractive-index mismatches across multilayer media, such as air, glass, and the electrolyte, thereby enabling more efficient excitation and collection of TERS signals with high sensitivity.^76^ To date, most EC–TERS implementations including the Ren group,^161,162^ van Duyne group,^159,160^ Kim group,^158^ and Domke group,^163,164^ have adopted side-illumination geometries for both excitation and collection of TERS signals, in combination with custom-designed STM heads with a large optical access and compact spectroelectrochemical cells. This configuration facilitates efficient light coupling to the tip–sample junction while accommodating the geometric constraints imposed by the electrochemical environment. In addition to side-illumination architectures, the Ren group also developed a top-illumination EC–AFM–TERS configuration.^165^ In this approach, metal-coated AFM–TERS probes were protected with an ultrathin SiO_2_ layer to enhance their mechanical robustness and chemical stability in liquid environments. Excitation and collection of the TERS signal were accomplished using a water-immersion objective positioned above the AFM probe. Further examples and applications of EC–TERS are discussed in section 3.2.3.

## 3 Application of TERS to Heterogeneous Catalysis Research

Heterogeneous catalytic reactions are inherently complex, typically comprising multiple elementary steps including adsorption of reactant molecules onto the catalyst surface, surface diffusion toward catalytically active sites, electron transfer between the surface and adsorbates, and chemical bond breaking and formation, followed by product desorption. Depending on whether the reaction is electrochemical or purely chemical in nature, these processes may involve transient reaction intermediates with short lifetimes and competing reaction pathways. The dynamic and multistep character of real catalytic reactions therefore presents a formidable challenge for experimental characterization, particularly at the nanoscale. Several TERS studies in catalysis have addressed this complexity by focusing on surface chemical transformations in model systems and on well-defined, idealized surfaces. Moreover, TERS investigations have predominantly been limited to 2D samples, although some cross-sectioning experiments have demonstrated the potential of TERS for 3D chemical characterization in real life catalytic materials.^70^ Nevertheless, the high sensitivity, nondestructive, label-free, and ambient-operability of TERS offers unique opportunities to probe catalytic processes at nanometer length scales in ways that are inaccessible to conventional analytical techniques.

In this section, we examine the literature reporting the application of TERS to heterogeneous catalysis. We begin with *ex situ* studies including investigations of organometallic porphyrin catalysts, bimetallic catalysts, supported Pt nanocatalysts, zeolite catalysts and Au(111) catalysts. We then discuss the rapidly expanding body of *in situ* and *operando* TERS studies, which we categorize into plasmon-driven photochemical reactions, hydrogenation reactions, and electrochemical reactions. Overall, photochemical and electrochemical TERS studies currently dominate the literature, likely reflecting the relative ease with which these reactions can be externally controlled through light illumination or applied potential, respectively.

### 3.1 *Ex Situ* TERS Studies

#### 3.1.1 Organometallic Porphyrin Catalysts

Metalloporphyrins and metallophthalocyanines have attracted considerable attention as catalysts in organic and electrochemical reactions owing to their high catalytic activity, well-defined active sites, and chemical stability. In the context of the oxygen reduction reaction (ORR), which is a key process in energy conversion technologies. Conventional catalysts typically rely on a high loading of precious metals such as Pt, which limits their economic and environmental sustainability. Metalloporphyrins and metallophthalocyanines are therefore regarded as promising alternative ORR catalysts. Depending on the pH, the ORR proceeds through distinct reaction pathways involving multiple surface-bound intermediates. TERS offers a unique opportunity to directly identify the coordination of small molecular and atomic species, such as O_2_, HO_2_^–^, OH^–^, and O, to the metal center with nanometer-scale spatial resolution.

In this area, an interesting TERS study was conducted by Domke and Pettinger,^166^ who investigated cobalt tetraphenylporphyrin (CoTPP) adlayers on Au(111), albeit outside the context of an active catalytic reaction. Their measurements revealed the coexistence of ordered CoTPP domains and spontaneously formed disordered phases. Importantly, the disordered regions exhibited additional TERS features attributed to axial CO and NO ligands coordinated to the Co center, whereas the ordered CoTPP adlayers showed no spectral signatures of axial ligand binding.

Additional contributions to this field were made by the Van Duyne group, who employed TERS to study cobalt and iron phthalocyanines (CoPc and FePc) on single-crystal metal substrates under UHV conditions as well as in electrochemical environments using EC–TERS (see section 3.2.3).^167^ In a particularly insightful study, Nguyen et al. used isotopic labeling with ^16^O_2_ and ^18^O_2_ to identify axial ligands bound to the Co center in a CoPc/Ag(111) system under UHV.^168^ Distinct TERS bands corresponding to different adsorption configurations of ^16^O_2_, ^18^O_2_, ^16^O, and ^18^O axial ligands were observed (Figure 5). The high spatial resolution of TERS was crucial for detecting these species, which would otherwise be obscured in ensemble-averaged measurements. Supported by density functional theory (DFT) calculations, the authors successfully assigned the band at 1151 cm^–1^ to an axial ^18^O_2_ ligand bound to CoPc (^18^O–^18^O stretching vibration), the band at 623 cm^–1^ to an axial ^18^O atom, and the band at 603 cm^–1^ to an axial ^16^O atom (Co–O stretching vibrations). The isotope-insensitive band at 661 cm^–1^ was attributed to an in-plane asymmetric distortion of the Pc ring. In these configurations, the Ag(111) substrate acted as the second axial ligand.

**Figure 5:**
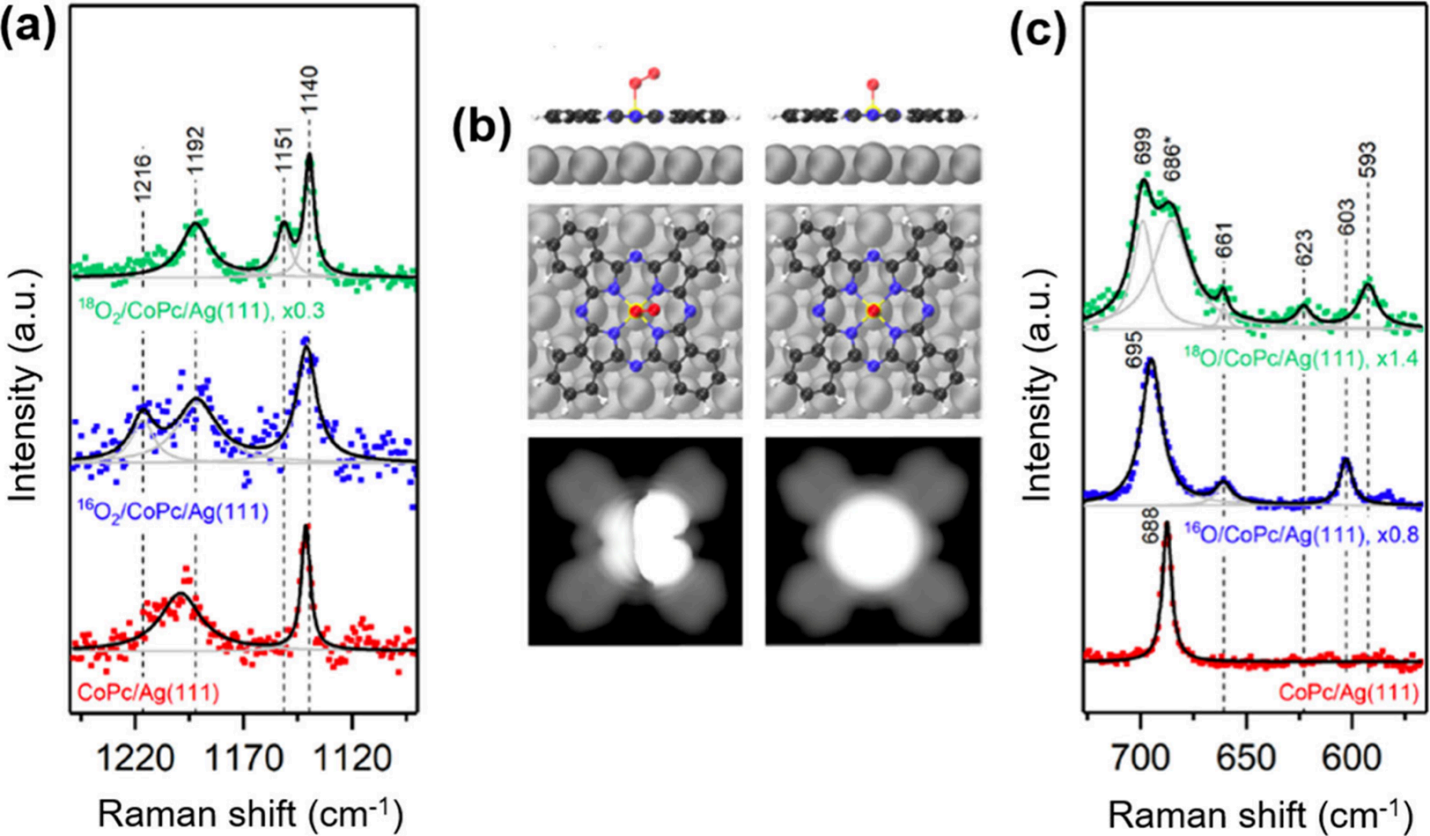
(a) Selected regions of UHV–TERS spectra acquired on CoPc/Ag(111) before O_2_ dosing (red) and after dosing with ^16^O_2_ (blue) and ^18^O_2_ (green) at O_2_/CoPc/Ag­(111) sites. (b) Calculated STM images and corresponding adsorption geometries for O_2_/CoPc/Ag­(111) and O/CoPc/Ag(111). Atom color code: H, white; C, black; N, blue; Co, yellow; Ag, gray; O, red. (c) Selected regions of UHV–TERS spectra acquired on O/CoPc/Ag(111) before O_2_ dosing (red) and after dosing with ^16^O_2_ (blue) and ^18^O_2_ (green). Adapted from ref 168. Copyright 2018 American Chemical Society.

#### 3.1.2 Bimetallic Catalysts

*Ex situ* studies of bimetallic catalysts have provided early but compelling demonstrations of the potential of TERS for nanoscale catalytic analysis, particularly in mapping the spatial distribution of active sites and reactive species. TERS enables direct visualization of interfacial effects that are central to the function of bimetallic catalytic systems. A representative example is the study by Yin et al.,^71^ who employed TERS to investigate hydrogen spillover from Pd onto an adjacent Au(111) surface (Figure 6a). Using chloro-nitrobenzenethiol (CNBT) as a Raman reporter molecule adsorbed on a well-defined Pd/Au(111) bimetallic surface, the authors monitored the selective hydrogenation of CNBT to chloro-aminobenzenethiol (CABT) upon exposure to H_2_. Under the reaction conditions, hydrogen dissociation occurred exclusively on Pd, while Au remained catalytically inactive. Postreaction TERS imaging revealed that hydrogenation extended beyond the Pd domains into neighboring Au regions. Quantitative analysis showed that reactive zones were located approximately 15–30 nm away from the Pd sites (Figure 6b–e), revealing the spatial extent of hydrogen spillover from Pd to Au.

**Figure 6:**
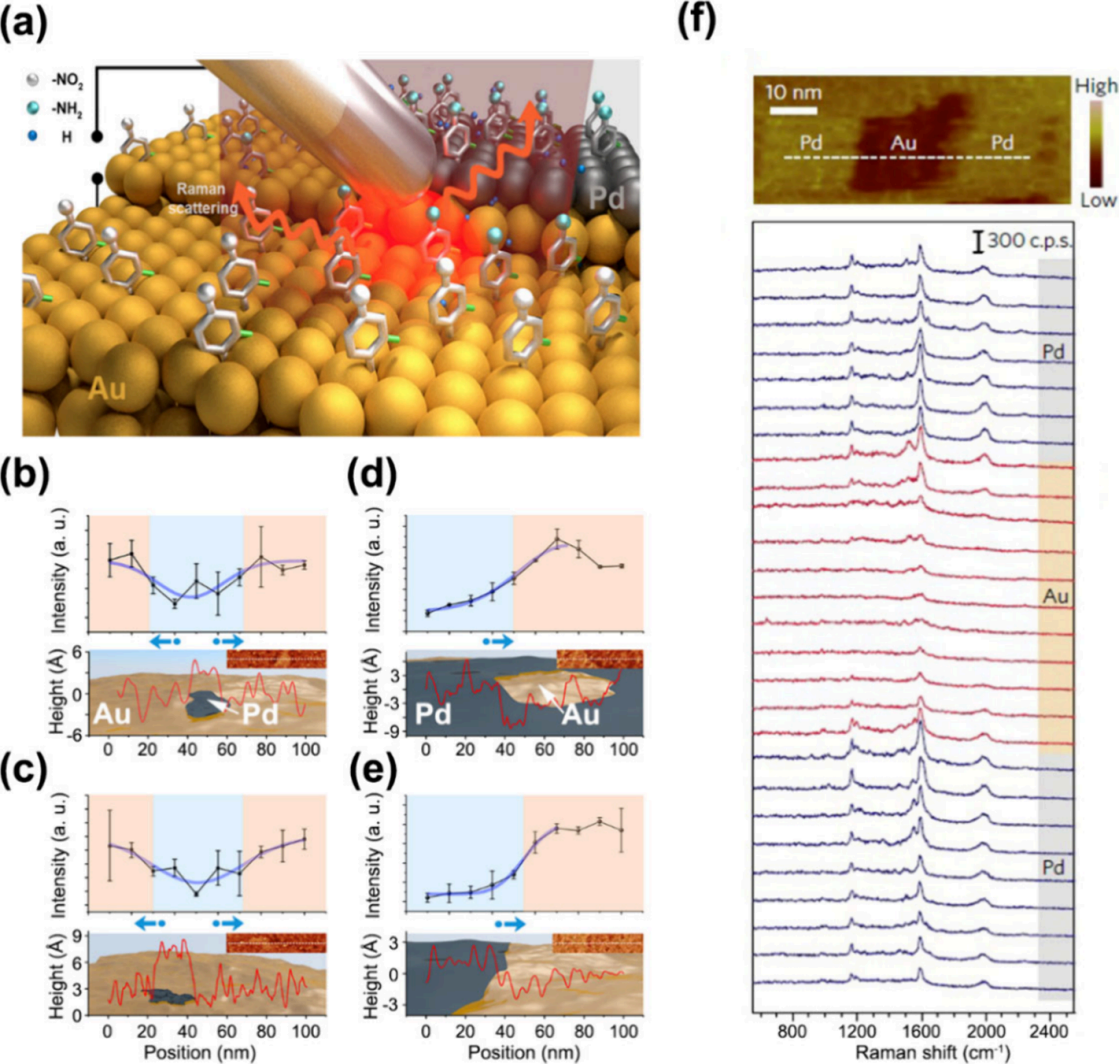
(a) Schematic illustration of an STM–TERS setup used to investigate hydrogenation of CNBT on a Pd/Au bimetallic substrate. Color code: nitro groups, silver; amino groups, cyan; C–Cl bonds, green; benzene rings, gray; hydrogen atoms, blue. (b,c) Intensity profiles of the 1336 cm^–1^ Raman band (associated with the nitro-group vibration) extracted from two representative TERS line scans acquired on a CNBT SAM supported on low-coverage Pd/Au after H_2_ exposure, together with the corresponding surface topography (red traces) obtained along the dashed lines indicated in the STM images. The intensity profiles are superimposed with schematics of the surface structure. (d,e) Corresponding TERS intensity profiles and topographic data for CNBT SAMs on high-coverage Pd/Au surfaces. In (b–e), blue shaded regions denote the reactive zones defined by the full width at half-maximum of the fitted curves (purple traces), and blue arrows indicate the direction of hydrogen spillover. (a–e) Adapted with permission from ref 71. Copyright 2020 Springer Nature. (f) STM topography and the corresponding TERS line-scan spectra of PIC adsorbed on a Pd/Au bimetallic surface acquired along the dashed line indicated in the STM image. (f) Adapted with permission from ref 170. Copyright 2017 Springer Nature.

A related approach was used to probe the generation and diffusion of reactive oxygen species on Pd/Au bimetallic surfaces.^169^ In this work, the thiolate 4-PBT served as a molecular probe on Pd/Au substrates. Exposure to H_2_O_2_ led to the formation of reactive oxygen species that oxidized and removed the thiolate from the surface. Subsequent TERS analysis demonstrated that Pd acted as the active site for oxygen species generation, with enhanced reactivity observed at Pd step edges relative to terrace sites, whereas Au remained largely inactive.

The relationship between the surface electronic properties and catalytic activity of a submonolayer Pd/Au(111) bimetallic surface was investigated by Ren and co-workers using oxidation of phenyl isocyanide (PIC).^170^ PIC was used as a probe molecule to detect the electronic and catalytic properties of the surface. It has been found that PIC can be oxidized to phenyl isocyanate on a Au(111) surface when exposed to air, whereas this reaction occurs less efficiently on a Pd surface. Moreover, the vibrational peak ν_(N=C)_ appears at a different Raman frequency when PIC is adsorbed on different metals (Au and Pd), due to its varied adsorption configurations on Au and on Pd surfaces. Based on the TERS line scans, the authors surprisingly discovered weaker NC bond signal and higher oxidation reactivity of PIC adsorbed at the Pd step edge compared to the Pd terraces (Figure 6f) due to the enhanced d−π* back-donation but decreased σ–d interaction. Finally, the authors demonstrated that the site-specific electronic and catalytic properties of a bimetallic surface can be spatially resolved with 3 nm spatial resolution, which is quite impressive for ambient STM–TERS. With such a high spatial resolution, the authors demonstrated that TERS is able to spatially distinguish vibrational features of molecules adsorbed at different surface sites, including defects and step edges, which play important roles in heterogeneous catalysis.

The same group investigated the site-specific electronic properties of a Pt nanoisland/Au(111) bimetallic surface.^171^ The Pt/Au bimetallic surface was prepared first, by Cu underpotential deposition on a Au(111) surface, followed by galvanic replacement by Pt. Using a similar probe molecule as before, 4-chloro-phenyl isocyanide (CPI), the authors were able to distinguish the electronic properties of the terrace, step edge, kink, and corner sites with different coordination environments on a 10 nm size Pt nanoisland with a spatial resolution of 2.5 nm. With the help of DFT calculations, they concluded that a lower coordination number at an atomic site leads to higher d-band center, which results in stronger metal-molecule interaction and a blue-shift of the ν_(N=C)_ peak of CPI molecules observed experimentally.

#### 3.1.3 Supported Pt Nanocatalysts

Coke formation on Pt nanorods supported on SiO_2_ following propane dehydrogenation was investigated using AFM-TERS by Filez et al.^172^ Postreaction analysis revealed pronounced chemical heterogeneity in the coke deposits, ranging from nanocrystalline graphite to disordered polymeric species. Notably, coke deposition on the Pt surface was highly nonuniform, with discrete hot spots enriched in disordered carbon appearing at isolated sites along the nanorods. Furthermore, coke was found to migrate from the Pt surface onto the SiO_2_ support, where it underwent further graphitization. The study offered guidelines for selectively regenerating coked metal surface and enhancing long-term catalyst stability.

#### 3.1.4 Fluid Cracking Catalysts

In addition to TERS, a closely related technique called tip-enhanced fluorescence (TEFL) microscopy, can be implemented within the same experimental setup and applied to investigate the activity of complex catalytic systems. In the early 20th century, the innovation of fluid catalytic cracking (FCC) transformed the petroleum industry by enabling higher gasoline yields and quality than thermal cracking.^173,174^ FCC converts long-chain hydrocarbons into shorter, more valuable products, typically using zeolite catalysts rich in Brønsted acid sites. However, catalyst deactivation during operation remains a major limitation, motivating nanoscale methods to resolve the distribution and evolution of active zeolite domains.

Traditionally, active domains have been visualized by reactive staining and confocal fluorescence microscopy, exploiting Brønsted acid–catalyzed thiophene oligomerization to generate fluorescent carbocationic products, though this approach is limited in sensitivity and spatial resolution.^175–178^ Kumar et al. overcame these limitations using TEFL microscopy on microtome-sectioned, spent FCC particles, enabling nanoscale mapping of acidity variations via the 650 and 700 nm emission bands associated with shorter and longer thiophene oligomers, respectively (Figure 7).^70^ The measurements revealed pronounced intraparticle heterogeneity, including broad domain-size distributions (0.002–0.28 μm^2^) and a trend toward smaller zeolite domains from the particle core to the edge. This work demonstrated the potential of TEFL microscopy for probing internal activity patterns in industrially relevant, 3D catalyst architectures, which can complement hyperspectral TERS imaging in nanoscale catalysis studies.

**Figure 7:**
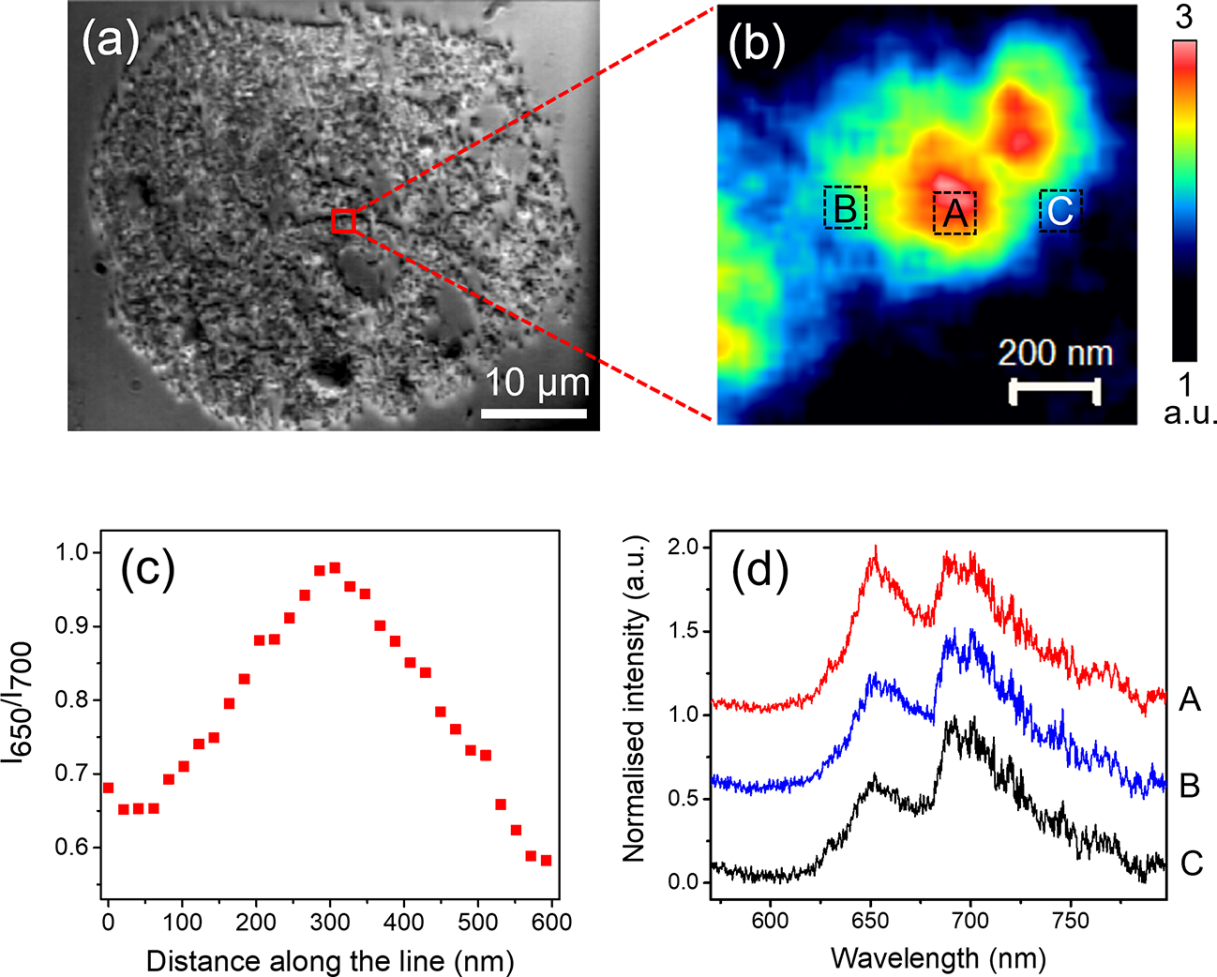
(a) Optical image of a 100 nm thick section of an industrially spent FCC particle mounted on a glass substrate. (b) TEFL chemical image obtained by integrating the intensity of the 650 and 700 nm TEFL bands over the 1 μm × 1 μm region highlighted in (a). Nominal step size, 20 nm; integration time, 1 s; laser power, 365 μW. (c) Spatial profile of the average intensity ratio of the 650 and 700 nm TEFL bands (*I*_650_/*I*_700_) from point B to point C in (b). (d) Averaged TEFL spectra acquired at positions A–C indicated in (b). Spectra are normalized and vertically offset for clarity. Reproduced with permission from ref 70. Copyright 2019 under the terms of the Creative Commons Attribution–NonCommercial–NoDerivatives 4.0 International (CC BY-NC-ND 4.0) license.

#### 3.1.5 Au­(111) Catalysts

TERS was used to probe the mechanism of O_2_ activation on an extended Au(111) surfaces by Cai et al.^179^ Mechanistic understanding of the activation of molecular oxygen on metal surfaces is pivotal for developing efficient catalysts for oxidative chemical reactions. Traditional analytical tools struggle with sensitivity and spatial resolution, making it challenging to empirically validate oxygen activation pathways. Cai et al. utilized TERS to overcome these limitations. Oxidative conversion of 4-ATP → 4-NBT in SAMs on Au(111) was employed as the model reaction system. Interestingly, oxidation was found to proceed more efficiently in disordered 4-ATP adlayers compared to ordered ones indicating that reaction occurs via interaction with on-surface oxidative species. Importantly, the study provided the first empirical evidence of water-promoted oxygen activation on extended Au(111) surfaces. TERS measurements of H_2_^18^O-treated 4-ATP SAM confirmed this mechanism, showing a red-shift in the ν­(NO_2_) signal of 4-NBT, indicative of the incorporation of ^18^O. These results provided the first empirical evidence of the molecular oxygen activation pathway on extended Au(111) surfaces.

Even though these *ex situ* studies focus on simplified model reactions and probe molecules, they establish important proof-of-principle demonstrations of how TERS can elucidate interfacial catalytic phenomena in bimetallic systems. With continued advances in probe stability, signal enhancement, and liquid-phase and *operando* TERS methodologies, extending such studies to more complex and industrially relevant catalytic reactions is expected to become increasingly feasible.

### 3.2 *In Situ* TERS Studies

#### 3.2.1 Plasmon-Driven Photocatalytic Reactions

Plasmon-driven photocatalytic reactions are the most extensively studied catalytic transformations by TERS because the strongly confined electromagnetic near-field in the tip–sample junction generates hot carriers that can initiate and steer surface chemistry with nanoscale selectivity. A prototypical and widely adopted model system is the plasmon-assisted coupling of 4-aminothiophenol (4-ATP) or 4-nitrobenzenethiol (4-NBT) to *p*,*p*′-dimercaptoazobenzene (DMAB).^180,181^ Owing to the large Raman cross sections of both reactants and product, this chemistry is spectroscopically straightforward to track, and it has therefore become a standard model reaction for interrogating plasmon-driven surface reactivity in SERS/TERS.

##### 3.2.1.1 Benchmark Coupling Chemistry: Tracking Time- and Spatially-Resolved Photocatalysis

The capability of TERS to monitor plasmon-driven photocatalysis *in situ* was first demonstrated by Weckhuysen and co-workers.^182^ Using bottom-illumination AFM–TERS, they initiated 4-NBT → DMAB conversion with 532 nm excitation by placing an Ag-coated probe in contact with a 4-NBT SAM on Au. To follow the reaction, they switched the excitation from 532 nm (initiation) to 633 nm (readout), enabling time-series TERS measurements that visualized photocatalytic molecular transformation localized at the probe apex.

TERS was subsequently advanced from point spectroscopy to imaging photocatalytic activity across heterogeneous surfaces. Kumar et al. demonstrated the first TERS imaging of a photocatalytic reaction on a heterogeneous catalyst surface by mapping the 4-ATP → DMAB reaction on patterned and rough Ag substrates with nanoscale resolution.^67^ Because Ag nanostructures are intrinsically active for this conversion, they passivated Ag-coated probes with 3–5 nm thick alumina layer using ALD (see section 2.5), rendering the probe chemically inert while retaining plasmonic enhancement. Using these inert probes, they imaged the spatial extent of the reaction on rough Ag with 20 nm resolution (Figure 8a–c), clearly distinguishing reactive “hotspots” from unreacted regions.

**Figure 8:**
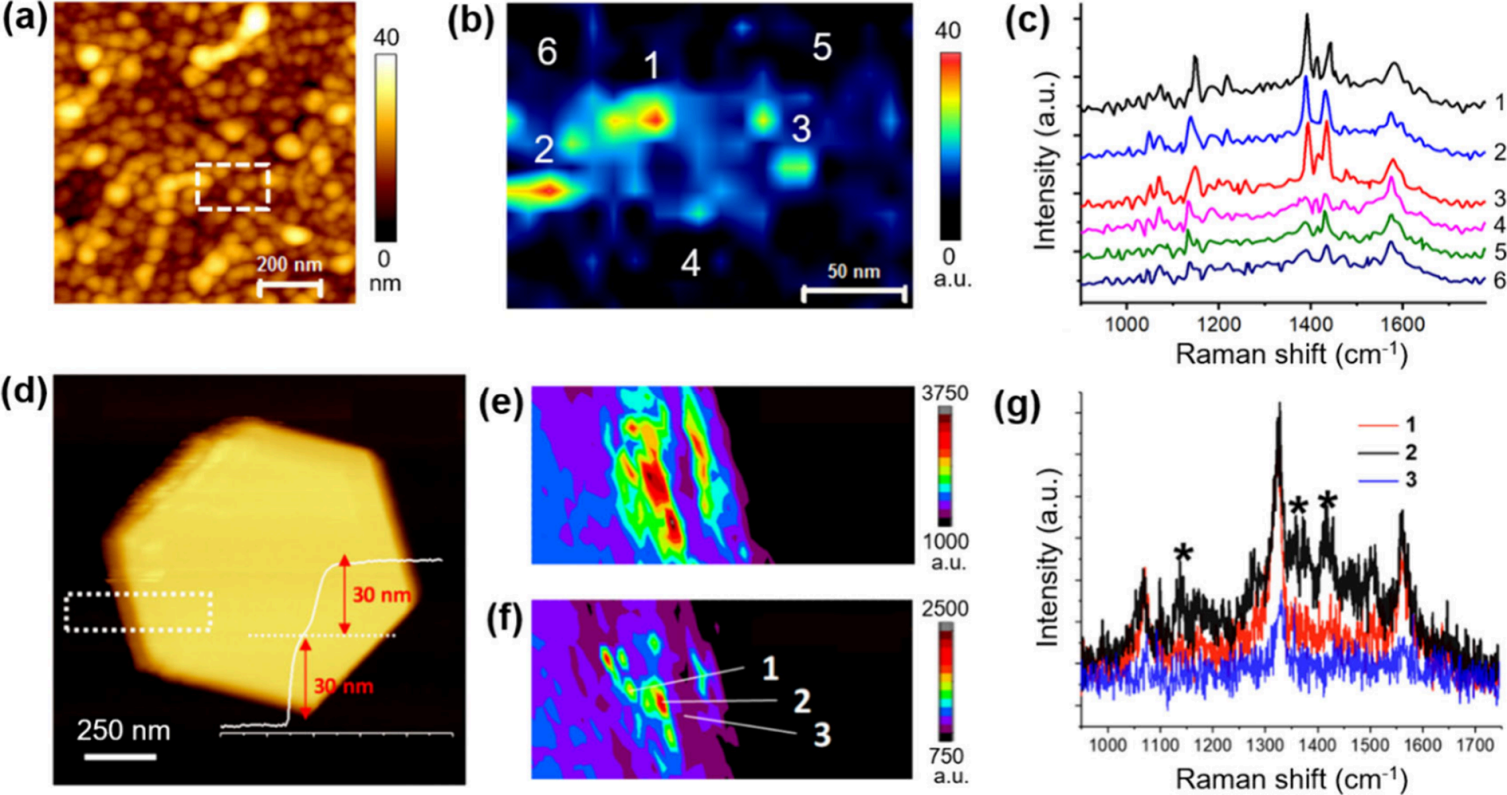
(a) AFM topography image of a rough Ag substrate. (b) TERS image of the 1142 cm^–1^ Raman band of DMAB acquired from the region highlighted in (a), revealing localized reaction hotspots associated with the 4-ATP → DMAB transformation. (c) Representative TERS spectra collected at reaction hotspots (positions 1–3) and away from the hotspots (positions 4–6) indicated in (b). (d) AFM topography image of a Au nanoplate. (e,f) TERS images of the characteristic Raman bands of 4-NBT and DMAB, respectively, acquired from the region highlighted in (d). (g) Representative TERS spectra collected at the positions indicated in (f), with stars marking the spectral positions of DMAB Raman bands. Notably, the spatial extent of DMAB formation is significantly smaller than that of the 4-NBT-functionalized region. (a–c) Adapted with permission from ref 67. Copyright 2015 The Royal Society of Chemistry. (d–g) Adapted from ref 183. Copyright 2019 American Chemical Society.

##### 3.2.1.2 Preserving Probe Functionality for Measurements in Liquids

A central methodological step toward tracking photocatalysis under realistic conditions is operation in liquid environments (section 2.5). To this end, zirconia-protected probes were developed using a simple, rapid wet-chemical method (section 2.4.3).^69^ These probes exhibited lifetimes exceeding 4.5 months, quantified via time-series TERS of PEDOT:PSS films, and showed markedly improved stability in liquids. Leveraging these advances, 4-ATP → DMAB reaction hotspots were imaged on rough Ag in aqueous solution with a spatial resolution of 14 nm, demonstrating that nanoscale *operando* mapping of plasmon-driven catalysis at solid–liquid interfaces is feasible when probe degradation is mitigated.^69^

##### 3.2.1.3 Mapping Local Reaction Landscape

Plasmon-driven transformations are often assumed to occur wherever the LSPR field is strongest, yet TERS revealed that the relationship between field enhancement and chemical conversion can be nontrivial. El-Khoury and co-workers interrogated whether all nanolocalized optical “hotspots” are equally competent sites for chemical transformation on plasmonic metals.^183^ They performed TERS imaging of 4-NBT-functionalized Au nanoplates in water using Au-coated probes in a bottom-illumination geometry. Using 633 nm excitation to initiate 4-NBT → DMAB on Au, they found that not all regions of high plasmonic enhancement produced DMAB to the same extent (Figure 8d–g). Notably, they observed *cis*-DMAB for the first time at the Au–water interface alongside *trans*-DMAB, and proposed that molecular crowding and steric constraints can strongly influence the local reaction outcome even within highly enhancing regions. In a related study, they detected an additional reaction channel during 4-NBT transformation.^184^ Using side-illumination TERS on 4-NBT-functionalized faceted Ag nanoparticles (Au-coated probes; 633 nm excitation for both initiation and monitoring), they observed anionic 4-NBT (1334 cm^–1^) more frequently than the conventional product DMAB and noted that the anionic species exhibit a stronger Raman cross section than 4-NBT and DMAB. These studies underscored that plasmon-driven coupling chemistry may proceed through multiple intermediates and that local environment and molecular organization can be decisive.

##### 3.2.1.4 Hot-Carrier Chemistry versus Plasmonic Heating

Disentangling hot-carrier pathways from photothermal effects remains a key question in plasmon-driven photocatalysis. 4-NBT → DMAB conversion has been shown to be a hot-carrier-driven reaction.^185,186^ Wang et al. examined 4-NBT-functionalized Au using side-illumination AFM–TERS with 671 nm excitation for both reaction initiation and monitoring.^187^ A fraction of spectra showed a thiolate feature (1305 cm^–1^), which could arise from either thermal desorption or hot-electron injection into the lowest unoccupied molecular orbital (LUMO) of 4-NBT. By combining experiments with finite-difference time-domain (FDTD)–finite element method (FEM) simulations, they estimated the near-field temperature rise to be only ∼305 K, which is insufficient for thermal desorption, thereby confirming the hot-electron mechanism for thiolate formation and reinforcing the nonthermal origin of the photochemistry in this system.

##### 3.2.1.5 Plasmon-Driven Photocatalysis on Materials beyond Au/Ag

TERS has also been used to monitor plasmon-driven photocatalysis on a broader range of substrates and catalytic materials. Li and Kurouski reported efficient 4-NBT → DMAB conversion on Cu nanowires and nanocubes, and further demonstrated plasmon-driven oxidation of 4-mercapto-phenyl-methanol (MPM) to 4-mercaptobenzoic acid (MBA) on Cu nanostructures, previously observed primarily on Au–Pt nanoplates.^188^ Patil and Kurouski subsequently showed that photocatalytic reduction of 4-NBT → DMAB can proceed on WS_2_ nanoplates supported on Si, with catalytic activity substantially enhanced upon functionalization with Pd nanoparticles.^189^ This highlights a promising direction in which catalytic metals and transition metal dichalcogenides can be integrated to boost plasmon-assisted reactivity.

##### 3.2.1.6 *In Situ* Reactivity and Selectivity on Bimetallic Surfaces

Bimetallic architectures offer a powerful testbed for understanding how composition modifies plasmonic fields, charge-carrier dynamics, and reaction selectivity at the nanoscale. Kurouski and co-workers used the 4-NBT dimerization reaction to interrogate Au/Pd microplates (Au@PdMPs) and pure Au microplates (AuMPs).^190^ TERS imaging revealed that Au@PdMPs catalyze 4-NBT to both DMAB and 4-ATP, whereas AuMPs yield DMAB exclusively. This product divergence was attributed to the dependence of reduction pathways on plasmon energy: strong electromagnetic fields favor DMAB formation, while weaker plasmon excitation favors 4-ATP. Because Pd exhibits interband transitions,^191^ plasmonic field damping occurs on Au@PdMPs but not on AuMPs, providing a mechanistic basis for the observed selectivity. Analogous selectivity was observed on Au@Pt nanoplates (Au@PtNPs), where 4-ATP was first oxidized to 4-NBT and was then reduced to DMAB, whereas 4-ATP on Au nanoplates underwent direct oxidation to DMAB.^192^ A limitation of the Au@PdMP study, however, was that the surface structure of the irregular Pd nanoclusters could not be resolved sufficiently to establish a definitive structure–reactivity correlation.

Beyond azo coupling, Li and Kurouski investigated redox selectivity on Au@Pd and Au@Pt nanoplates and showed that MPM → MBA oxidation proceeds exclusively on Au@Pt, whereas MBA → MPM reduction occurs exclusively on Au@Pd.^193^ They also observed C–C cleavage of both MPM and MBA on Au nanoplates, yielding thiophenol. In a further extension, they examined plasmon-driven Suzuki–Miyaura coupling on Au@Pd nanoplates and found that coupling efficiency decreases systematically from 4-bromo- to 4-chloro- to 4-fluoro-thiophenols (4-BTP> 4-CTP> 4-FTP), enabling rapid nanoscale ranking of bimetallic catalytic reactivity.^194^

##### 3.2.1.7 Structure–Reactivity Relationship

Although plasmon-enhanced coupling reactions are widely used as model systems,^68^ the role of reactant molecular arrangement in determining reaction efficiency has only recently been clarified. Cai et al. combined nanoscale STM–TERS imaging with molecular-resolution ambient STM and DFT modeling to interrogate how reactant organization controls photocatalytic coupling of 4-NBT → DMAB on single-crystal and polycrystalline Au.^119^ By comparing “drop-cast” and “immersion” preparation protocols, they established that disordered, kinetically trapped 4-NBT adlayers exhibit significantly higher coupling efficiency than thermodynamically stable ordered phases. TERS imaging (3 nm resolution) and DFT analysis linked the reduced reactivity of ordered phases to steric hindrance and high energy barriers, establishing an unambiguous structure–reactivity relationship for plasmon-driven on-surface coupling reactions. These insights were supported by Dong and co-workers, who investigated coverage-dependent 4-NBT → DMAB dimerization on Ag(100) and Ag(111) using single-molecule TERS under UHV cryogenic conditions.^195^ The reaction was suppressed in densely packed monolayers due to steric hindrance and occurred only at submonolayer coverage; moreover, direct contact between 4-NBT and the Ag substrate was essential, confirming the critical role of plasmon-driven charge transfer in DMAB formation.

##### 3.2.1.8 Mechanistic understanding of Photocatalytic Coupling and Reverse Reactions

Complementary STM–TERS work has also sharpened mechanistic understanding of the coupling reaction and its reverse process. Sun et al. employed a home-built high-vacuum STM–TERS system to study plasmon-driven dimerization of 4-NBT.^185^ Despite the practical difficulty of combining laser optics with HV instrumentation, they achieved acceptable signal-to-noise TERS spectra using a Au tip, noting that an unprotected Au probe may itself contribute catalytically. Increasing laser intensity from 10 μW to 2 mW led to complete spectral conversion from 4-NBT to DMAB, consistent with higher hot-carrier densities promoting coupling. Furthermore, increasing the tunneling-current set point at constant bias reduced the NO_2_ band intensity (1336 cm^–1^), consistent with a reduced gap and stronger LSPR. They excluded a dominant photothermal contribution because the nanogap temperature did not significantly change when the tunneling current was increased at constant bias,^186^ likely because thermal energy dissipates efficiently along the tip and/or substrate. They also ruled out contributions from photoelectron emission from the probe or substrate, as no DMAB signal was observed when the tip was retracted even at 10 mW excitation. Later, Ren and co-workers showed that dimerization of 4-NBT or 4-ATP does not proceed on Ag(111) but can occur on Au(111), attributing this to distinct adsorption geometries on Ag(111) that hinder coupling; by contrast, rough Ag surfaces and nanoparticles permit multiple orientations, enabling reaction.^196^ This suggests that the Ag-coated films used in Sun’s study were likely rough.^185^

Sun et al. further investigated the reverse reaction, DMAB dissociation, activated by hot carriers.^197^ Using *ex situ* TERS under HV, they showed that dissociation products depend strongly on environment: under acidic conditions (pH = 3), 4-ATP formed via protonation of the radical fragment; under alkaline conditions (pH = 11), 4-NBT formed, attributed to bonding of oxygen ions (presumably hydroxide) to the radical fragment. Under neutral conditions, no dissociation-product Raman signals were detected due to rapid reformation of DMAB. They proposed three requirements for selectively observing dissociation products: sustained hot-carrier generation (“plasmonic scissors”) from strong LSPR, availability of H^+^/O^2–^ species to stabilize radical fragments, and sufficiently weak plasmons during readout to avoid recoupling while acquiring product spectra.

##### 3.2.1.9 Plasmonic Hotspots As Reactive Nanoreactors

Beyond azo coupling, surface plasmons can also drive other reactions such as O_2_ and H_2_ dissociation,^198,199^ and in many cases plasmon-induced transformations are undesired side processes during TERS, manifesting as spurious spectral features. Szczerbiński et al. performed an in-depth SERS and TERS study of plasmon-driven chemistry by monitoring the transformation of seven thiolate SAMs on Au as laser intensity was increased.^200^ They observed products were strikingly similar to those formed in surface photochemistry induced by secondary electrons under X-ray or electron-beam irradiation. By analyzing the anti-Stokes background, they distinguished hot-electron energy distributions and quantified hotspot temperature rises (300–480 K for 0.04–2.37 mW), supporting photocatalytic, not purely thermal origins of the chemistry. They also observed amorphous carbon formation upon desorption and fragmentation and proposed that mechanisms familiar from electron-beam studies under UHV, such as desorption induced by electronic transitions, can be reproduced in SERS and TERS hotspots under ambient conditions. Extending this framework to biomolecules, they showed that increased laser intensity can dissociate peptide backbone bonds, evidenced by disappearance of the amide I band, and that the resulting products resemble those generated by electron capture dissociation and electron transfer dissociation in the gas phase.^201^ Although these studies are not conventional catalytic reactions, they raise an important interpretive question: what transformations are induced within the plasmonic hotspot as laser power is increased, and how should these be distinguished from the intended chemistry? Because hot-carrier-driven decomposition is often uncontrolled and undesirable, careful optimization of excitation intensity is essential to minimize unintended sample transformation during TERS measurements.

##### 3.2.1.10 Tracking Photocatalysis at Single-Bond Level

Dong and co-workers pushed plasmon-driven photocatalysis to its ultimate spatial limit by demonstrating that STM–TERS can resolve reactions at the single chemical-bond level.^202^ Using subnanometer-resolved STM–TERS under UHV, they tracked bond breaking and formation within an individual chemisorbed molecule by monitoring laser-induced dehydrogenation and hydrogen-transfer reactions of an up-standing melamine molecule on Cu(100), with a vertical detection depth of ∼4 Å. This study highlights the extraordinary potential of STM–TERS for interrogating elementary photocatalytic reaction steps with unprecedented chemical specificity and spatial resolution.

#### 3.2.2 Pt-Catalyzed Hydrogenation

Hydrogenation of nitroarenes on Pt is a prototypical yet mechanistically intricate heterogeneous reaction, where ensemble-averaged approaches often obscure site-specific kinetics and transient surface states.^203^ In our recent study, we addressed this gap by implementing *in situ* STM-TERS to follow the hydrogenation of CNTP to CATP directly at a single plasmonic hotspot on a well-defined Pt(111) surface under ambient conditions with continuous H_2_ flow.^204^ Time-sequenced spectra (1 s integration) captured the real-time decay of CNTP marker modes and the concomitant emergence of CATP signatures, revealing a characteristic reaction time scale of ∼6–7 s. Critically, control measurements on CNTP/Au(111) showed no spectral changes under identical illumination and H_2_ exposure, demonstrating that the transformation requires surface H generated by H_2_ dissociation on Pt rather than laser heating or plasmon-generated hot carriers. The *in situ* approach was essential because it disentangled catalytic hydrogenation from concurrent adsorbate dynamics: overall TERS intensity decrease was linked to hydrogen-induced cleavage of Pt–S bonds and partial CNTP desorption during reaction. By correlating the dynamic TERS measurements with first-principle DFT calculations, CATP was identified as the dominant product (excluding significant C–Cl reduction), and the kinetic bottleneck was assigned to the second H-addition step (0.83 eV barrier; ∼10 s), consistent with the experimentally observed seconds-scale dynamics. This work illustrates how *in situ* TERS, integrated with DFT, can deliver site-specific, time-resolved mechanistic insights on a nonplasmonic catalyst surface, providing a practical framework for extending *operando* nanospectroscopy to realistic hydrogenation catalysis.

#### 3.2.3 Electrochemical TERS

Considerable interest has emerged in the development of EC–TERS for probing charge-transfer processes at solid–liquid interfaces, which play a central role in many catalytic reactions. In EC–TERS, the probe and sample are immersed in an electrolyte together with reference and counter electrodes, with the sample serving as the working electrode. Application of an external potential enables precise control over electron-transfer rates between the electrode and molecular species in solution or adsorbed at the surface, providing a powerful platform for *in situ* investigation of surface electrocatalytic transformations with nanoscale spatial resolution.

Unlike other electrochemical techniques such as EC-SERS, which typically reports an ensemble-averaged response from a comparatively large interfacial area^205^ and attenuated total reflection surface-enhanced infrared absorption spectroscopy (SEIRAS) or normal SEIRAS, which offer excellent surface sensitivity for IR-active intermediates but still averages over the illuminated electrode area,^206^ EC–TERS uniquely provides molecular “fingerprint” information with nanoscale spatial resolution at the electrified solid–liquid interface under working conditions. Likewise, while scanning electrochemical microscopy can map local electrochemical activity with high spatial resolution providing mechanistic insights, it does not provide direct vibrational identification of specific adsorbates or intermediates;^207^ EC–TERS complements this by delivering chemically specific, spatially resolved vibrational spectra that help establish local structure–activity relationships and elucidate electrocatalytic mechanisms. The nanoscale probe volume inherent to EC–TERS is key to the identification of spatially localized and transient interfacial species, mapping of potential-dependent adsorption configurations, and direct correlation of local reactivity with surface heterogeneities.

Although relatively few EC–TERS studies have addressed catalysis in the strictest sense, they have nevertheless yielded valuable insights into interfacial chemical reactions, thereby advancing our understanding of electrocatalytic processes and surface reaction mechanisms. Importantly, they have also established a robust methodological foundation for the application of TERS to catalysis under electrochemical control. The first demonstrations of EC–TERS were reported in 2015 by the Ren and Van Duyne groups.^152,208^ Ren and co-workers implemented EC–TERS by introducing the excitation light horizontally into an EC–STM cell and investigating the potential-dependent behavior of a monolayer of 4-PBT adsorbed on Au(111) in 0.1 M NaClO_4_.^152^ Subtle, yet reproducible, changes in the TERS spectra were observed revealing variations in the protonation state of 4-PBT as a function of applied potential. Shortly thereafter, the Van Duyne group reported EC–TERS measurements in AFM mode, enabling direct observation of redox transformations of Nile Blue (NB) adsorbed on an indium tin oxide (ITO) surface.^208^ In this work, “TERS voltammograms” were constructed by plotting the intensities of Raman bands associated with the oxidized and reduced forms of NB as a function of electrode potential. The step-like features observed in these plots were interpreted as signatures of the small number of molecules probed within the TERS near-field.

Since these pioneering studies, both the Ren and Van Duyne groups continued to expand the scope of EC–TERS, demonstrating its applicability across a range of electrochemical systems. Notably, EC–TERS has been employed to investigate the ORR catalyzed by organometallic porphyrin molecules.^75,160^ The Van Duyne group applied EC–TERS to study CoPc and FePc at the solid–liquid interface on Au(111) under conditions where the ORR can take place. They identified a highly ordered monolayer of CoPc molecules at potentials larger than 0.1 V (vs. a reversible hydrogen electrode), while there was a phase transition to a diffusion phase at <0.1 V, accompanied by the disappearance of the TERS signals.^75^ Their study demonstrated that partially reduced CoPc molecules are the dominant species under steady state measurements during the ORR. In another study, they used EC–TERS for *operando* characterization of FePc as a model catalyst for the ORR.^160^ This study revealed both reversible changes as well as irreversible degradation of the FePc during the course of the reaction. The irreversible degradation was interpreted to be a consequence of FePc demetalation and the formation of the free base phthalocyanine via a direct loss of Fe^2+^. Mechanistically, it was important to acquire evidence that catalyst deactivation is not a consequence of carbon corrosion.

A number of other research groups have subsequently demonstrated EC–TERS measurements in STM mode, most commonly applied to potential-induced chemical changes in molecular monolayers adsorbed on Au electrodes.^158,163,209^ From the perspective of catalysis, the primary interest lies in reactions occurring directly at electrode surfaces; the discussion below therefore focuses on such surface-confined processes. Mattei et al. extended the concept of TERS voltammetry to quantitatively map spatial variations in the formal redox potential of NB across ITO surfaces, demonstrating that EC–TERS can interrogate adsorbates at or near the single-molecule limit.^210^ Supported by quantitative modeling, they proposed that the electrochemical behavior of the neutral (reduced) form of NB is less sensitive to the local environment than that of the cationic (oxidized) form, highlighting the capability of EC–TERS to probe intermolecular interactions and local electrochemical heterogeneity at surfaces. In a subsequent study, the same group employed EC–TERS imaging to investigate Au(111) nanoplates supported on ITO, again using the NB redox couple as a model system.^211^ This work revealed pronounced spatial contrast between different materials and enabled resolution of site-specific variations in the formal redox potential across individual ITO grains, with a reported spatial resolution of 40 nm.

More directly related to electrocatalysis, Pfisterer et al. used EC–TERS imaging to study the site-specific electro-oxidation of Au(111) electrodes in 0.1 M H_2_SO_4_ (Figure 9a).^164^ Surface oxidation was found to occur at less anodic potentials on defect sites compared to atomically flat Au terraces (Figure 9b), reflecting the enhanced electrocatalytic activity of defects toward water activation. The oxidation process was monitored *in situ* by tracking the appearance of the Au oxide Raman band at 560–580 cm^–1^, which was present at 1.45 V (vs Pd–H) but absent at 1.10 V (Figure 9c). Correlation of EC–TERS intensity maps with surface topography (Figure 9d) unambiguously demonstrated that oxidation was localized exclusively at defect sites. The authors further identified two distinct oxide species, attributing Au_2_O_3_ formation primarily to flatter defect-terrace features and Au_2_O to sharper protrusions. Knowledge of such structure–activity relationships is critical to the development of improved catalysts and in this case, it is envisaged that new insights may follow into the oxygen evolution reaction, which is the kinetic bottleneck in water electrolysis.

**Figure 9:**
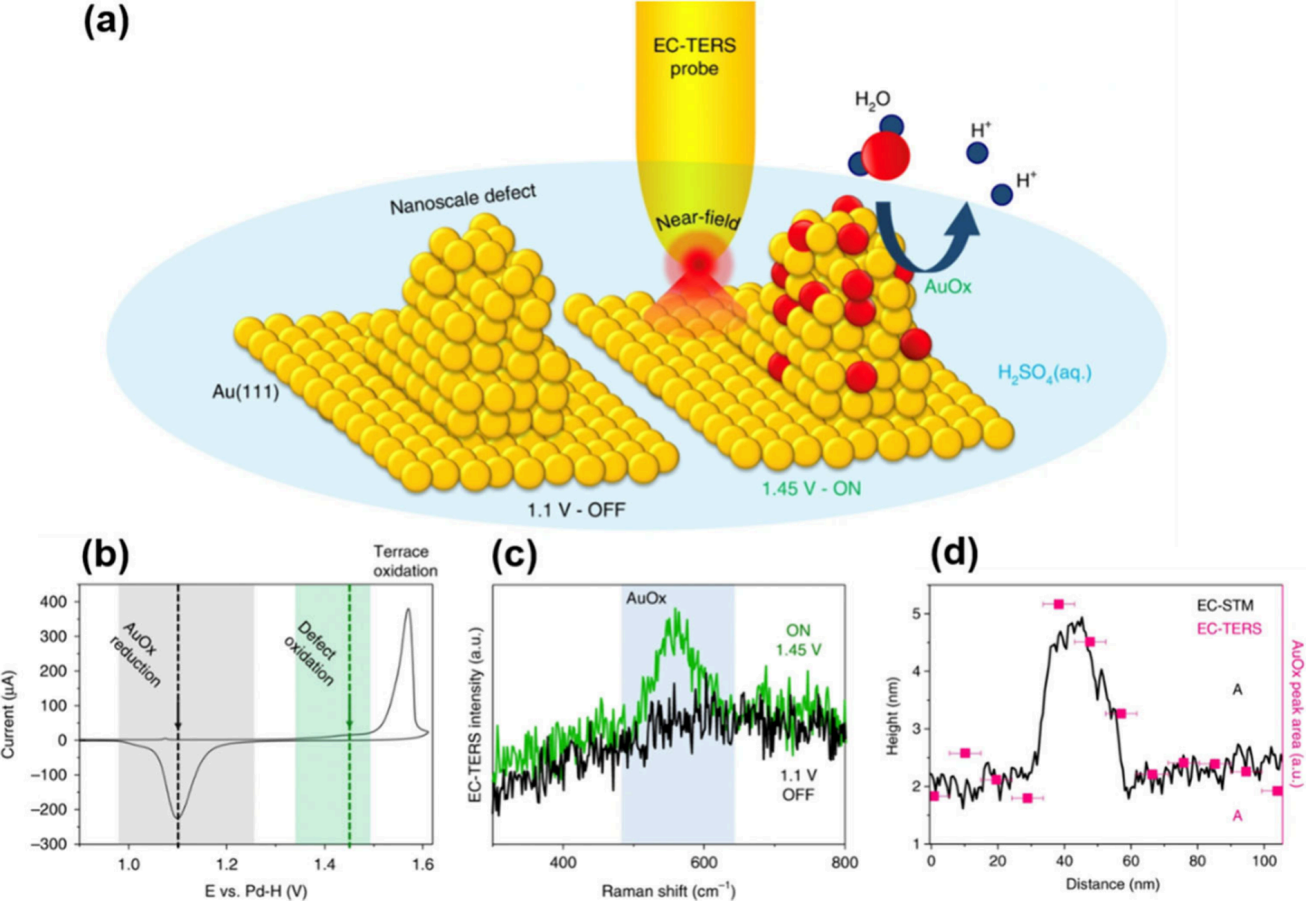
(a) Schematic illustration of the EC–TERS configuration used to investigate oxidation processes at Au surface defects. (b) Cyclic voltammogram of Au(111) in 0.1 M H_2_SO_4_ recorded at a scan rate of 50 mV s^–1^, highlighting the potential regions associated with defect oxidation and Au oxide reduction. (c) EC–TERS spectra showing the presence of the Au oxide Raman band at an active defect site at 1.45 V vs Pd–H and its absence at 1.10 V. (d) Correlation between the STM topography line profile (solid line, left axis) and the corresponding EC–TERS intensity of the Au oxide band (square symbols, right axis) recorded across an isolated Au defect. Adapted with permission from ref 164. Copyright 2019 under the terms of the Creative Commons Attribution (CC BY) license.

Recently, Fiocco et al. used EC–TERS to study the electrochemical reduction of 4-NBT SAMs on Au electrodes. Formation of the DMAB, produced frequently in the photocatalytic reactions of 4-ATP and 4-NBT (see section 3.2.1), was observed under potentiostatic and potentiodynamic conditions.^212^ Related to this, Huang and co-workers explored the electrochemical reduction of 4-nitrophenyl isocyanide on Pd/Au(111) surfaces using EC–TERS.^162^ With the aid of atomistic modeling, they were able to isolate a negatively charged anionic intermediate in neutral solution, validating the notion of a stepwise electron transfer followed by protonation under these conditions.

As already discussed in section 3.2.1, plasmon-driven catalytic reactions have been the focus of a significant number of TERS studies. A key feature of these studies is that the localized surface plasmons excited in the probe–sample junction, which gives rise to TERS enhancement, can simultaneously drive the surface reaction. In cases where independent excitation of the probe and sample is not feasible, it becomes experimentally challenging to perform control measurements in which TERS spectra are acquired in the absence of a surface reaction. EC–TERS addresses this limitation by enabling modulation of the sample Fermi level through an applied potential bias, thereby allowing plasmon-driven reactions to be selectively switched on or off. Huang et al. demonstrated this notion for the photocatalytic decarboxylation of 4-mercaptobenzoic acid molecules, adsorbed on Au(111) in 0.1 M NaClO_4_ (pH 10) solution.^161^ The reaction was characterized by an attenuation of the carboxylate stretching vibration in the TERS spectrum at 1400 cm^–1^ with concomitant growth of bands at 998 cm^–1^ and 1020 cm^–1^, attributed to the decarboxylation product, thiophenol. Using EC–TERS, it was demonstrated that the reaction could be modulated by application of a voltage bias to the sample, and at more positive potentials the energy of the photoinduced holes in the electrode was found to be sufficient to oxidize OH^–^ to •OH radicals, which then initiated the decarboxylation reaction. Furthermore, by virtue of the high spatial resolution of EC–TERS, the transport distance of the photogenerated holes (or hot carriers) through Au could also be measured.

Recently, Huang et al. employed EC–TERS to investigate the structural evolution of active sites in MoS_2_ during the electrocatalytic hydrogen evolution reaction (HER).^213^ By monitoring individual active sites under potential control, they were able to directly track geometric and electronic changes in MoS_2_ during the HER. EC–TERS imaging revealed reconstructed regions on the order of 40 nm, characterized by variations in lattice structure and electron density extending from the edge toward the basal plane. The study showed that initially disordered edge sites undergo progressive activation during the HER, leading to reduced activation barriers and enhanced electrocatalytic activity. Importantly, this work provided the first direct visualization of active-site dynamics in MoS_2_ under electrochemical conditions, demonstrating how edge reconstruction and lattice deformation promote catalytic performance.

Over the past decade, EC–TERS has evolved from a proof-of-principle technique into a powerful approach for probing mechanistic, spatially resolved electrochemical processes with molecular specificity at working solid–liquid interfaces. Early EC–TERS established that interfacial vibrational fingerprints can be tracked *in situ* as a function of electrode potential, enabling direct identification of potential-dependent adsorption/charge-state changes in surface-bound molecules at the nanoscale.^152^ Building on this, EC–TERS has delivered *operando* structure–activity information on molecular electrocatalysts, for example by directly monitoring changes in FePc under oxygen-reduction conditions and linking spectral evolution to catalyst deactivation pathways that are otherwise difficult to isolate in ensemble electrochemistry.^160^ A major conceptual advance is that EC–TERS can quantify nanoscale electrochemical kinetics/thermodynamics via “TERS voltammetry” and redox mapping: potential-dependent spectral switching of a reporter molecule such as NB allows extraction of local formal potentials and reveals spatial heterogeneity in redox behavior across different electrode materials and nanostructures.^210,211^ Importantly, EC–TERS has also enabled reactivity mapping under electrochemical turnover, pinpointing how nanoscale defects or specific sites dominate activity.^164^ Beyond electrochemical reactivity, EC–TERS uniquely bridges electrochemistry and plasmonics by providing real-space visualization of plasmon-generated hot-carrier chemistry and showing that carrier transport lengths and reactivity depend on carrier energy, insights that directly inform nanoscale device/reactor design for plasmon-assisted electrocatalysis.^161^ Furthermore, recent EC–TERS studies demonstrate genuine intermediate-level mechanistic resolution of key charged intermediates in nitro-derivative electroreduction and track the structural evolution of individual catalytic active sites during electrocatalytic hydrogen evolution, highlighting EC–TERS as a route to correlate transient interfacial chemistry with dynamic active-site reconstruction rather than static pre/post characterization.^162,213^

## 4 Conclusions and Outlook

### 4.1 Methodology

TERS is a highly sensitive, nondestructive, and label-free molecular imaging technique that provides nanoscale spatial resolution, making it uniquely suited for advancing the fundamental understanding of heterogeneous catalysis. As discussed in this article, continuous methodological developments, particularly in probe design, stability, and environmental compatibility, have enabled the successful application of TERS to an increasing number of catalytic systems.^66,69,144^ Despite these advances, TERS remains an emerging technique in catalysis research, with both strengths and further development opportunities, as summarized in Table 2.

**Table 2:** Current Capabilities and Future Development Opportunities for Application of TERS to Heterogeneous Catalysis

current capabilities	development opportunities
nanoscale spatial resolution under ambient conditions	current imaging times are long (20–60 min)
single molecule sensitivity	only surface characterization possible
molecular and surface specificity	low yield and reproducibility of TERS probes
correlative topography information	absolute quantification currently not possible
operation in both air and liquid environments	*operando* measurements are being developed
can be coupled with other SPM modes to provide other properties (e.g., electrical, mechanical etc.) simultaneously	limited application to highly rough and/or porous materials

Recent progress in high-performance TERS probe engineering has substantially reduced several critical barriers for catalytic applications, notably in terms of fabrication yield, chemical inertness, and operational stability. High probe yield has been demonstrated by tailoring the dielectric environment of metal-coated AFM tips using thin dielectric films or thermally grown oxide layers prior to metallization,^125,126^ while pulsed or potentiostatic electrodeposition has improved control over coating morphology and probe-to-probe reproducibility.^127,128^ For catalysis-relevant measurements, ultrathin dielectric passivation layers (e.g., silica, alumina and zirconia) have enabled chemically inert Ag probes that retain strong near-field enhancement, facilitating nanoscale mapping of catalytic activity without perturbing surface chemistry.^67^ In parallel, significant extensions of plasmonic probe lifetime have been demonstrated using alumina and zirconia protective layers.^69,149^ Further progress toward measurements at solid–liquid interfaces includes the development of liquid-compatible coatings, adhesion layers, and robust multilayer metallization strategies that mitigate delamination and enable stable TERS imaging in aqueous environments.^66,73,153^ In addition, reproducible electrochemically etched Au probes have enabled reliable EC–TERS measurements under potential control.^133,152^

Under ambient conditions, TERS routinely achieves spatial resolutions of a few nanometers and, with appropriate probe preparation, can be extended to chemically relevant liquid environments. As a result, nanoscale imaging of catalytic reactions under operating conditions is increasingly within reach. However, realizing this ambition still requires overcoming substantial experimental challenges. For reactions in aggressive liquid environments, further improvements in probe robustness and lifetime are essential. For gas-phase catalysis, both the probe and sample must be integrated into specially designed reaction cells capable of operating at elevated temperatures and pressures with controlled gas flow, while still permitting efficient optical excitation and Raman signal collection. Successful implementation of such designs would mark a decisive step toward *operando* TERS measurements of solid catalysts.

Most TERS studies reported to date have focused on steady-state systems, avoiding the additional complexity associated with time-dependent chemical processes. In contrast, real catalytic systems are inherently dynamic, and access to real-time nanoscale information would provide transformative insights into reaction mechanisms and catalyst evolution. Achieving this goal critically depends on improving data acquisition speed, which remains a major limitation of current TERS imaging. While SPM techniques are generally slower than optical microscopies, recent advances in video-rate AFM^214^ suggest viable pathways toward faster TERS measurements, potentially enabling time-resolved monitoring of catalytic reactions in the future.

Despite notable progress in the commercialization of TERS instrumentation, performing reliable and reproducible TERS imaging remains challenging. Beyond issues such as plasmonic tip degradation and sample instability under intense localized electromagnetic fields, maintaining precise optical alignment in AFM–TERS systems remains a major experimental challenge, particularly during long-duration measurements. To address this limitation, Kato et al. demonstrated an effective drift-compensation strategy that actively corrects both lateral tip drift and axial focus drift with nanometer precision.^215^ By combining laser-scanning–assisted tip tracking with real-time focus stabilization, this approach enables continuous hyperspectral TERS imaging over several hours without loss of laser–tip alignment or degradation of signal intensity in either lateral or vertical dimensions.

### 4.2 Applications

Extending the applicability of TERS to industrially relevant catalytic processes requires robust strategies. To date, the majority of TERS studies have focused on idealized model systems, such as self-assembled monolayers covalently bound to atomically flat substrates via thiol linkers. While these systems have provided valuable mechanistic insights, they differ from practical catalysts, which are typically hierarchical, 3D composites with complex morphology and composition. This discrepancy highlights the inherently surface-sensitive nature of TERS, which complicates direct interrogation of bulk or porous catalytic materials.

The integration of TERS with advanced sample preparation techniques offers promising routes to overcome this limitation. For example, microtome sectioning has been used to expose internal surfaces of industrial catalysts, enabling nanoscale mapping of catalytic activity inside FCC particles using TEFL imaging.^70^ This approach demonstrates a viable pathway toward applying TERS to structurally complex, real-world catalysts.

Although TERS has evolved substantially as an analytical technique since its inception, its application to heterogeneous catalysis remains a largely unexplored and highly promising research frontier. As emphasized in this article, extending TERS to structurally and compositionally complex catalysts under realistic reaction conditions is an active area of development. Continued advances in probe engineering, instrumentation, and experimental strategies are steadily expanding the scope of TERS. Consequently, TERS is increasingly establishing itself as a powerful nanoanalytical tool capable of delivering molecularly specific, spatially resolved insights into catalytic systems under realistic conditions.

## References

[1] Rothenberg, G. Catalysis: Concepts and Green Applications, 2nd ed.; Wiley-VCH, Weinheim, 2008.

[2] Scott, R. A. Encyclopedia of Inorganic and Bioinorganic Chemistry; John Wiley & Sons, Ltd., 2011.

[3] Taseska T., Yu W., Wilsey M. K., Cox C. P., Meng Z., Ngarnim S. S., Müller A. M. (2023). Analysis of the Scale of Global Human Needs and Opportunities
for Sustainable Catalytic Technologies. Top
Catal..

[4] Chorkendorff, I. ; Niemantsverdriet, J. W. Concepts of Modern Catalysis and Kinetics; Wiley-VCH, Weinheim, 2017.

[5] Lavacchi, A. ; Miller, H. ; Vizza, F. Nanotechnology in Electrocatalysis for Energy; Springer-Verlag: New York, 2013.

[6] Wexler, P. Encyclopedia of Toxicology, 3rd ed.; Academic Press: Oxford, 2014.

[7] Weckhuysen, B. M. In Situ Spectroscopy of Catalysts; American Scientific Publishers: Stevenson Ranch, 2004.

[8] Ryczkowski J. (2001). IR spectroscopy
in catalysis. Catal. Today.

[9] Buurmans I. L. C., Weckhuysen B. M. (2012). Heterogeneities
of individual catalyst
particles in space and time as monitored by spectroscopy. Nat. Chem..

[10] Hunger M., Weitkamp J. (2001). In situ IR, NMR, EPR, and UV/Vis Spectroscopy: Tools
for New Insight into the Mechanisms of Heterogeneous Catalysis. Angew. Chem., Int. Ed..

[11] Nuñez J., Renslow R., Cliff J. B., Anderton C. R. (2018). NanoSIMS for biological
applications: Current practices and analyses. Biointerphases.

[12] Devaraj A., Perea D. E., Liu J., Gordon L. M., Prosa T. J., Parikh P., Diercks D. R., Meher S., Kolli R. P., Meng Y. S. (2018). Three-dimensional nanoscale characterisation of materials
by atom probe tomography. Int. Mater. Rev..

[13] Wiktor C., Meledina M., Turner S., Lebedev O. I., Fischer R. A. (2017). Transmission
electron microscopy on metal–organic frameworks – a
review. J. Mater. Chem. A.

[14] Kurouski D., Dazzi A., Zenobi R., Centrone A. (2020). Infrared and Raman
chemical imaging and spectroscopy at the nanoscale. Chem. Soc. Rev..

[15] Bossers K.
W., Valadian R., Zanoni S., Smeets R., Friederichs N., Garrevoet J., Meirer F., Weckhuysen B. M. (2020). Correlated
X-ray Ptychography and Fluorescence Nano-Tomography on the Fragmentation
Behavior of an Individual Catalyst Particle during the Early Stages
of Olefin Polymerization. J. Am. Chem. Soc..

[16] Wang Z., Tang Y., Zhang L., Li M., Shan Z., Huang J. (2020). In Situ TEM Observations of Discharging/Charging
of Solid-State Lithium-Sulfur
Batteries at High Temperatures. Small.

[17] Wang L., Xie R., Chen B., Yu X., Ma J., Li C., Hu Z., Sun X., Xu C., Dong S., Chan T.-S., Luo J., Cui G., Chen L. (2020). In-situ visualization of the space-charge-layer
effect on interfacial lithium-ion transport in all-solid-state batteries. Nat. Commun..

[18] Stockle R. M., Suh Y. D., Deckert V., Zenobi R. (2000). Nanoscale chemical
analysis by tip-enhanced Raman spectroscopy. Chem. Phys. Lett..

[19] Anderson M. S. (2000). Locally
enhanced Raman spectroscopy with an atomic force microscope. Appl. Phys. Lett..

[20] Hayazawa N., Inouye Y., Sekkat Z., Kawata S. (2000). Metallized Tip Amplification
of Near-field Raman Scattering. Opt. Commun..

[21] Pettinger B., Picardi G., Schuster R., Ertl G. (2000). Surface enhanced Raman
spectroscopy: Towards single molecular spectroscopy. Electrochemistry.

[22] Kumar N., Mignuzzi S., Su W., Roy D. (2015). Tip-enhanced Raman
spectroscopy: principles and applications. EPJ.
Technol. Instrum..

[23] Kumar N., Weckhuysen B. M., Wain A. J., Pollard A. J. (2019). Nanoscale chemical
imaging using tip-enhanced Raman spectroscopy. Nat. Protoc..

[24] Xie X. S., Hartschuh A., Sanchez E. J., Novotny L. (2003). High-resolution Near-field
Raman Microscopy of Single Walled Carbon Nanotubes. Phys. Rev. Lett..

[25] Cançado L. G., Jorio A., Ismach A., Joselevich E., Hartschuh A., Novotny L. (2009). Mechanism of Near-Field Raman Enhancement
in One-Dimensional Systems. Phys. Rev. Lett..

[26] Tschannen C. D., Vasconcelos T. L., Novotny L. (2022). Tip-enhanced Raman spectroscopy of
confined carbon chains. J. Chem. Phys..

[27] Yoshikawa M., Murakami M., Fujita Y. (2022). Structural
characterization of intersections
between semiconducting and metallic carbon nanotubes using tip-enhanced
Raman spectroscopy. J. Raman Spectrosc..

[28] Stadler J., Schmid T., Zenobi R. (2011). Nanoscale
Chemical Imaging of Single-Layer
Graphene. ACS Nano.

[29] Pollard A. J., Kumar N., Rae A., Mignuzzi S., Su W., Roy D. (2014). Nanoscale optical spectroscopy:
an emerging tool for the characterization
of graphene and related 2-D Materials. J. Mater.
Nanosci..

[30] Mignuzzi S., Kumar N., Brennan B., Gilmore I. S., Richards D., Pollard A. J., Roy D. (2015). Probing individual
point defects
in graphene via near-field Raman scattering. Nanoscale.

[31] Su W., Kumar N., Dai N., Roy D. (2016). Nanoscale mapping of
intrinsic defects in single-layer graphene using tip-enhanced Raman
spectroscopy. Chem. Commun..

[32] Su W., Kumar N., Krayev A., Chaigneau M. (2018). In situ topographical
chemical and electrical imaging of carboxyl graphene oxide at the
nanoscale. Nat. Commun..

[33] Legge E. J., Paton K. R., Wywijas M., McMahon G., Pemberton R., Kumar N., Aranga Raju A. P., Dawson C. P., Strudwick A. J., Bradley J. W. (2020). Determining
the Level and Location of Functional
Groups on Few-Layer Graphene and Their Effect on the Mechanical Properties
of Nanocomposites. ACS Appl. Mater. Interfaces..

[34] Su W., Esfandiar A., Lancry O., Shao J., Kumar N., Chaigneau M. (2021). Visualising
structural modification of patterned graphene
nanoribbons using tip-enhanced Raman spectroscopy. Chem. Commun..

[35] Miranda H., Rabelo C., Cançado L. G., Vasconcelos T. L., Oliveira B. S., Schulz F., Lange H., Reich S., Kusch P., Jorio A. (2020). Impact of substrate on tip-enhanced
Raman spectroscopy: A comparison between field-distribution simulations
and graphene measurements. Physical Review Research.

[36] Yoshikawa M., Murakami M., Fujita Y. (2022). Characterization
of inhomogeneity
at edges of graphene oxide films using tip-enhanced Raman spectroscopy. J. Raman Spectrosc..

[37] Su W., Kumar N., Spencer S. J., Dai N., Roy D. (2015). Transforming
bilayer MoS2 into single-layer with strong photoluminescence using
UV-ozone oxidation. Nano Res..

[38] Su W., Kumar N., Mignuzzi S., Crain J., Roy D. (2016). Nanoscale
mapping of excitonic processes in single-layer MoS2 using tip-enhanced
photoluminescence microscopy. Nanoscale.

[39] Su W., Kumar N., Shu H., Lancry O., Chaigneau M. (2021). In Situ Visualization
of Optoelectronic Behavior of Grain Boundaries in Monolayer WSe2 at
the Nanoscale. J. Phys. Chem. C.

[40] Shao J., Chen F., Su W., Kumar N., Zeng Y., Wu L., Lu H.-W. (2022). Probing
Nanoscale Exciton Funneling at Wrinkles of
Twisted Bilayer MoS2 Using Tip-Enhanced Photoluminescence Microscopy. J. Phys. Chem. Lett..

[41] Lee C., Jeong B. G., Yun S. J., Lee Y. H., Lee S. M., Jeong M. S. (2018). Unveiling Defect-Related Raman Mode of Monolayer WS2
via Tip-Enhanced Resonance Raman Scattering. ACS Nano.

[42] Kato R., Umakoshi T., Sam R. T., Verma P. (2019). Probing nanoscale defects
and wrinkles in MoS2 by tip-enhanced Raman spectroscopic imaging. Appl. Phys. Lett..

[43] Sam R. T., Umakoshi T., Verma P. (2020). Probing stacking
configurations in
a few layered MoS2 by low frequency Raman spectroscopy. Sci. Rep..

[44] Lee C., Jeong B. G., Kim S. H., Kim D. H., Yun S. J., Choi W., An S.-J., Lee D., Kim Y.-M., Kim K. K., Lee S. M., Jeong M. S. (2022). Investigating
heterogeneous
defects in single-crystalline WS2 via tip-enhanced Raman spectroscopy. npj 2D Materials and Applications.

[45] Krayev A., Isotta E., Hoang L., Yang J. A., Neilson K., Wang M., Haughn N., Pop E., Mannix A., Balogun O. (2025). Excitation Laser Energy
Dependence of the Gap-Mode
TERS Spectra of WS2 and MoS2 on Silver. ACS
Photonics.

[46] Zheng L.-Q., Servalli M., Schlüter A. D., Zenobi R. (2019). Tip-enhanced Raman
spectroscopy for structural analysis of two-dimensional covalent monolayers
synthesized on water and on Au (111). Chem.
Sci..

[47] Zhang Z., Liu H., Sun Q., Shao F., Pan Q., Zhuang T., Zhao Y. (2020). Interfacial
Synthesis of a Monolayered Fluorescent Two-Dimensional
Polymer through Dynamic Imine Chemistry. ChemistryOpen.

[48] Kumar N., Zoladek-Lemanczyk A., Guilbert A. A. Y., Su W., Tuladhar S. M., Kirchartz T., Schroeder B. C., McCulloch I., Nelson J., Roy D., Castro F. A. (2017). Simultaneous topographical,
electrical and optical microscopy of optoelectronic devices at the
nanoscale. Nanoscale.

[49] Wang X., Zhang D., Braun K., Egelhaaf H. J., Brabec C. J., Meixner A. J. (2010). High-resolution spectroscopic mapping of the chemical
contrast from nanometer domains in P3HT:PCBM organic blend films for
solar-cell applications. Adv. Funct. Mater..

[50] Kumar N., Drozdz M. M., Jiang H., Santos D. M., Vaux D. J. (2017). Nanoscale
mapping of newly-synthesised phospholipid molecules in a biological
cell using tip-enhanced Raman spectroscopy. Chem. Commun..

[51] Kumar, N. Amino Acids, Peptides and Proteins; The Royal Society of Chemistry: London, 2019; Vol. 43.

[52] Stepanenko T., Sofińska K., Wilkosz N., Dybas J., Wiercigroch E., Bulat K., Szczesny-Malysiak E., Skirlińska-Nosek K., Seweryn S., Chwiej J. (2024). Surface-enhanced Raman
scattering (SERS) and tip-enhanced Raman scattering (TERS) in label-free
characterization of erythrocyte membranes and extracellular vesicles
at the nano-scale and molecular level. Analyst.

[53] Pandey Y., Kumar N., Goubert G., Zenobi R. (2021). Nanoscale Chemical
Imaging of Supported Lipid Monolayers using Tip-Enhanced Raman Spectroscopy. Angew. Chem., Int. Ed..

[54] Wang J., Jiang Z., He Z., Zhang P., Yi Z., Sokolov A. V., Scully M. O. (2025). Tip-enhanced Raman spectroscopy of
cell wall heterogeneity for Aspergillus Fumigatus. Appl. Phys. Lett..

[55] Yeo B. S., Amstad E., Schmid T., Stadler J., Zenobi R. (2009). Nanoscale
probing of a polymer-blend thin film with tip-enhanced Raman spectroscopy. Small.

[56] Mrđenović D., Abbott D., Mougel V., Su W., Kumar N., Zenobi R. (2022). Visualizing Surface Phase Separation in PS-PMMA Polymer
Blends at the Nanoscale. ACS Appl. Mater. Interfaces..

[57] Agapov R. L., Scherger J. D., Sokolov A. P., Foster M. D. (2015). Identification of
individual isotopes in a polymer blend using tip enhanced Raman spectroscopy. J. Raman Spectrosc..

[58] Zheng L.-Q., Wang X., Shao F., Hegner M., Zenobi R. (2018). Nanoscale
Chemical Imaging of Reversible Photoisomerization of an Azobenzene-Thiol
Self-Assembled Monolayer by Tip-Enhanced Raman Spectroscopy. Angew. Chem., Int. Ed..

[59] Zheng L.-Q., Yang S., Lan J., Gyr L., Goubert G., Qian H., Aprahamian I., Zenobi R. (2019). Solution Phase and
Surface Photoisomerization of a Hydrazone Switch with a Long Thermal
Half-Life. J. Am. Chem. Soc..

[60] Zheng L. Q., Yang S., Krähenbühl S., Rybkin V. V., Lan J., Aprahamian I., Zenobi R. (2022). Effect of the alkyl linker length
on the photoisomerization of hydrazone switches on metal surfaces. Materials Today Chemistry.

[61] Tallarida N., Rios L., Apkarian V. A., Lee J. (2015). Isomerization
of One
Molecule Observed through Tip-Enhanced Raman Spectroscopy. Nano Lett..

[62] Bartolomeo G. L., Zhang Y., Kumar N., Zenobi R. (2021). Molecular
Perturbation
Effects in AFM-Based Tip-Enhanced Raman Spectroscopy: Contact versus
Tapping Mode. Anal. Chem..

[63] Cai Z.-F., Zheng L.-Q., Zhang Y., Zenobi R. (2021). Molecular-Scale Chemical
Imaging of the Orientation of an On-Surface Coordination Complex by
Tip-Enhanced Raman Spectroscopy. J. Am. Chem.
Soc..

[64] Hong M., Yokota Y., Hayazawa N., Kazuma E., Kim Y. (2020). Homogeneous
Dispersion of Aromatic Thiolates in the Binary Self-Assembled Monolayer
on Au(111) via Displacement Revealed by Tip-Enhanced Raman Spectroscopy. J. Phys. Chem. C.

[65] Wang X., Zhong J.-H., Zhang M., Liu Z., Wu D.-Y., Ren B. (2016). Revealing intermolecular interaction
and surface restructuring of
an aromatic thiol assembling on Au (111) by tip-enhanced Raman spectroscopy. Analytical chemistry.

[66] Kumar N., Su W. T., Vesely M., Weckhuysen B. M., Pollard A. J., Wain A. J. (2018). Nanoscale chemical imaging of solid-liquid
interfaces using tip-enhanced Raman spectroscopy. Nanoscale.

[67] Kumar N., Stephanidis B., Zenobi R., Wain A. J., Roy D. (2015). Nanoscale
mapping of catalytic activity using tip-enhanced Raman spectroscopy. Nanoscale.

[68] Hartman T., Wondergem C. S., Kumar N., van den Berg A., Weckhuysen B. M. (2016). Surface- and Tip-Enhanced Raman Spectroscopy in Catalysis. J. Phys. Chem. Lett..

[69] Kumar N., Wondergem C. S., Wain A. J., Weckhuysen B. M. (2019). In Situ
Nanoscale Investigation of Catalytic Reactions in the Liquid Phase
Using Zirconia-Protected Tip-Enhanced Raman Spectroscopy Probes. J. Phys. Chem. Lett..

[70] Kumar N., Kalirai S., Wain A. J., Weckhuysen B. M. (2019). Nanoscale
Chemical Imaging of a Single Catalyst Particle with Tip-Enhanced Fluorescence
Microscopy. ChemCatChem..

[71] Yin H., Zheng L.-Q., Fang W., Lai Y.-H., Porenta N., Goubert G., Zhang H., Su H.-S., Ren B., Richardson J. O. (2020). Nanometre-scale spectroscopic visualization
of catalytic sites during a hydrogenation reaction on a Pd/Au bimetallic
catalyst. Nat. Catal..

[72] Huang S.-C., Bao Y.-F., Wu S.-S., Huang T.-X., Sartin M. M., Wang X., Ren B. (2021). Electrochemical
Tip-Enhanced Raman
Spectroscopy: An In Situ Nanospectroscopy for Electrochemistry. Annu. Rev. Phys. Chem..

[73] Schmid T., Yeo B. S., Leong G., Stadler J., Zenobi R. (2009). Performing
tip-enhanced Raman spectroscopy in liquids. J. Raman Spectrosc..

[74] Touzalin T., Dauphin A. L., Joiret S., Lucas I. T., Maisonhaute E. (2016). Tip enhanced
Raman spectroscopy imaging of opaque samples in organic liquid. Phys. Chem. Chem. Phys..

[75] Jiang S., Chen Z., Chen X., Nguyen D., Mattei M., Goubert G., Van Duyne R. P. (2019). Investigation of Cobalt Phthalocyanine
at the Solid/Liquid Interface by Electrochemical Tip-Enhanced Raman
Spectroscopy. J. Phys. Chem. C.

[76] Huang S. C., Ye J. Z., Shen X. R., Zhao Q. Q., Zeng Z. C., Li M. H., Wu D. Y., Wang X., Ren B. (2019). Electrochemical
Tip-Enhanced Raman Spectroscopy with Improved Sensitivity Enabled
by a Water Immersion Objective. Anal. Chem..

[77] Willets K. A., Van Duyne R. P. (2007). Localized
surface plasmon resonance spectroscopy and
sensing. Annu. Rev. Phys. Chem..

[78] Kawata, S. ; Shalaev, V. M. Tip Enhancement; Elsevier: Amsterdam, 2007.

[79] Liao P. F., Wokaun A. (1982). Lightning rod effect in surface enhanced
Raman scattering. J. Chem. Phys..

[80] Vasconcelos T. L., Archanjo B. S., Oliveira B. S., Silva W. F., Alencar R. S., Rabelo C., Achete C. A., Jorio A., Cançado L. G. (2021). Optical
Nanoantennas for Tip-Enhanced Raman Spectroscopy. IEEE J. Sel. Top. Quantum Electron..

[81] Cirera B., Litman Y., Lin C., Akkoush A., Hammud A., Wolf M., Rossi M., Kumagai T. (2022). Charge Transfer-Mediated
Dramatic Enhancement of Raman Scattering upon Molecular Point Contact
Formation. Nano Lett..

[82] Yang B., Chen G., Ghafoor A., Zhang Y.-F., Zhang X.-B., Li H., Dong X.-R., Wang R.-P., Zhang Y., Zhang Y., Dong Z.-C. (2023). Chemical
Enhancement and Quenching in Single-Molecule
Tip-Enhanced Raman Spectroscopy. Angew. Chem.,
Int. Ed..

[83] Latorre F., Kupfer S., Bocklitz T., Kinzel D., Trautmann S., Gräfe S., Deckert V. (2016). Spatial resolution of tip-enhanced
Raman spectroscopy – DFT assessment of the chemical effect. Nanoscale.

[84] Sun M., Fang Y., Yang Z., Xu H. (2009). Chemical and electromagnetic
mechanisms of tip-enhanced Raman scattering. Phys. Chem. Chem. Phys..

[85] Cui X., Zhang W., Yeo B. S., Zenobi R., Hafner C., Erni D. (2007). Tuning the resonance frequency of Ag-coated dielectric tips. Opt. Express.

[86] Verma P. (2017). Tip-enhanced
Raman spectroscopy: Technique and recent advances. Chem. Rev..

[87] Meng L., Huang T., Wang X., Chen S., Yang Z., Ren B. (2015). Gold-coated AFM tips for tip-enhanced
Raman spectroscopy: theoretical
calculation and experimental demonstration. Opt. Express.

[88] Zhang W., Cui X., Martin O. J. F. (2009). Local field enhancement of an infinite conical metal
tip illuminated by a focused beam. J. Raman
Spectrosc..

[89] Goncharenko A. V., Wang J.-K., Chang Y.-C. (2006). Electric near-field enhancement of
a sharp semi-infinite conical probe: Material and cone angle dependence. Phys. Rev. B.

[90] Huang T.-X., Huang S.-C., Li M.-H., Zeng Z.-C., Wang X., Ren B. (2015). Tip-enhanced Raman spectroscopy:
tip-related issues. Anal. Bioanal. Chem..

[91] Sheremet E., Rodriguez R. D., Zahn D. R. T., Milekhin A. G., Rodyakina E. E., Latyshev A. V. (2014). Surface-enhanced Raman scattering and gap-mode tip-enhanced
Raman scattering investigations of phthalocyanine molecules on gold
nanostructured substrates. J. Vac. Sci. Technol.
B.

[92] He L., Rahaman M., Madeira T. I., Zahn D. R. T. (2021). Understanding
the Role of Different Substrate Geometries for Achieving Optimum Tip-Enhanced
Raman Scattering Sensitivity. Nanomaterials.

[93] Zhang J., Youssef A. H., Dörfler A., Kolhatkar G., Merlen A., Ruediger A. (2020). Sample induced intensity
variations
of localized surface plasmon resonance in tip-enhanced Raman spectroscopy. Opt. Express.

[94] Zhang R., Zhang Y., Dong Z. C., Jiang S., Zhang C., Chen L. G., Zhang L., Liao Y., Aizpurua J., Luo Y. (2013). Chemical mapping of a single molecule by plasmon-enhanced
Raman scattering. Nature.

[95] Lee J., Crampton K. T., Tallarida N., Apkarian V. A. (2019). Visualizing vibrational
normal modes of a single molecule with atomically confined light. Nature.

[96] Zhang Y., Yang B., Ghafoor A., Zhang Y., Zhang Y.-F., Wang R.-P., Yang J.-L., Luo Y., Dong Z.-C., Hou J. G. (2019). Visually constructing the chemical
structure of a single
molecule by scanning Raman picoscopy. Natl.
Sci. Rev..

[97] Zenobi R., Kumar N., Verma P. (2025). Spatial Resolution
in Nanoscale TERS
Imaging: Current Status, Challenges, and Guidelines. Nano Lett..

[98] Richard-Lacroix M., Zhang Y., Dong Z., Deckert V. (2017). Mastering
high resolution
tip-enhanced Raman spectroscopy: towards a shift of perception. Chem. Soc. Rev..

[99] Blum C., Opilik L., Atkin J. M., Braun K., Kammer S. B., Kravtsov V., Kumar N., Lemeshko S., Li J. F., Luszcz K. (2014). Tip-enhanced Raman spectroscopy - an interlaboratory
reproducibility and comparison study. J. Raman
Spectrosc..

[100] Bartolomeo G. L., Goubert G., Zenobi R. (2020). Tip Recycling for Atomic
Force Microscopy-Based Tip-Enhanced Raman Spectroscopy. Appl. Spectrosc..

[101] Zhang D., Wang X., Braun K., Egelhaaf H. J., Fleischer M., Hennemann L., Hintz H., Stanciu C., Brabec C. J., Kern D. P. (2009). Parabolic mirror-assisted
tip-enhanced spectroscopic imaging for non-transparent materials. J. Raman Spectrosc..

[102] Jalili N., Laxminarayana K. (2004). A review of atomic force microscopy
imaging systems: application to molecular metrology and biological
sciences. Mechatronics.

[103] Kuk Y., Silverman P. (1989). Scanning tunneling
microscope instrumentation. Rev. Sci. Instrum..

[104] Bailo E., Deckert V. (2008). Tip-enhanced Raman
spectroscopy of
single RNA strands: Towards a novel direct-sequencing method. Angew. Chem., Int. Ed..

[105] Yu J., Saito Y., Ichimura T., Kawata S., Verma P. (2013). Far-field
free tapping-mode tip-enhanced Raman microscopy. Appl. Phys. Lett..

[106] Umakoshi T., Kawashima K., Moriyama T., Kato R., Verma P. (2022). Tip-enhanced Raman
spectroscopy with amplitude-controlled tapping-mode
AFM. Sci. Rep..

[107] Rickman R. H., Dunstan P. R. (2014). Enhancement of lattice defect signatures
in graphene and ultrathin graphite using tip-enhanced Raman spectroscopy. J. Raman Spectrosc..

[108] Li L., Schultz J. F., Mahapatra S., Lu Z., Zhang X., Jiang N. (2022). Chemically identifying single adatoms with single-bond sensitivity
during oxidation reactions of borophene. Nat.
Commun..

[109] Li L., Mahapatra S., Schultz J. F., Zhang X., Jiang N. (2025). Single-molecule
spectroscopic probing of N-heterocyclic carbenes on a two-dimensional
metal. Chem..

[110] Kazemi-Zanjani N., Vedraine S., Lagugné-Labarthet F. (2013). Localized
enhancement of electric field in tip-enhanced Raman spectroscopy using
radially and linearly polarized light. Opt.
Express.

[111] Hayazawa N., Saito Y., Kawata S. (2004). Detection and characterization
of longitudinal field for tip-enhanced Raman spectroscopy. Appl. Phys. Lett..

[112] Lerman G. M., Levy U. (2008). Effect of radial polarization and
apodization on spot size under tight focusing conditions. Opt. Express.

[113] Wang, J. ; Wu, X. ; Wang, R. ; Zhang, M. Electronic Properties of Carbon Nanotubes; InTech: London, 2011.

[114] GarciaVidal F. J., Pendry J. B. (1996). Collective theory
for surface enhanced
Raman scattering. Phys. Rev. Lett..

[115] Mauser N., Hartschuh A. (2014). Tip-enhanced
near-field optical microscopy. Chem. Soc. Rev..

[116] Kumar N., Rae A., Roy D. (2014). Accurate measurement
of enhancement factor in tip-enhanced Raman spectroscopy through elimination
of far-field artefacts. Appl. Phys. Lett..

[117] Armstrong R. E., van Liempt J. C., Zijlstra P. (2019). Effect of Film Thickness
on the Far- and Near-Field Optical Response of Nanoparticle-on-Film
Systems. J. Phys. Chem. C.

[118] Shao F., Dai W., Zhang Y., Zhang W., Schlüter A. D., Zenobi R. (2018). Chemical Mapping of
Nanodefects within
2D Covalent Monolayers by Tip-Enhanced Raman Spectroscopy. ACS Nano.

[119] Cai Z.-F., Merino J. P., Fang W., Kumar N., Richardson J. O., De Feyter S., Zenobi R. (2022). Molecular-Level Insights
on Reactive Arrangement in On-Surface Photocatalytic Coupling Reactions
Using Tip-Enhanced Raman Spectroscopy. J. Am.
Chem. Soc..

[120] Shannon C. E. (1949). Communication
in the Presence of Noise. Proceedings of the
IRE.

[121] Heymann J. B., Möller C., Müller D. J. (2002). Sampling
effects influence heights measured with atomic force microscopy. J. Microsc..

[122] Zhang M., Wang R., Zhu Z., Wang J., Tian Q. (2013). Experimental research on the spectral
response of tips for tip-enhanced
raman spectroscopy. J. Opt..

[123] Leiterer C., Wünsche E., Singh P., Albert J., Köhler J. M., Deckert V., Fritzsche W. (2016). High precision
attachment of silver nanoparticles on AFM tips by dielectrophoresis. Anal. Bioanal. Chem..

[124] Walke P., Fujita Y., Peeters W., Toyouchi S., Frederickx W., De Feyter S., Uji-i H. (2018). Silver nanowires for
highly reproducible cantilever based AFM-TERS microscopy: towards
a universal TERS probe. Nanoscale.

[125] Yeo B. S., Schmid T., Zhang W., Zenobi R. (2007). Towards rapid
nanoscale chemical analysis using tip-enhanced Raman spectroscopy
with Ag-coated dielectric tips. Anal. Bioanal.
Chem..

[126] Hayazawa N., Yano T., Kawata S. (2012). Highly reproducible
tip-enhanced Raman scattering using an oxidized and metallized silicon
cantilever tip as a tool for everyone. J. Raman
Spectrosc..

[127] Yang L.-K., Huang T.-X., Zeng Z.-C., Li M.-H., Wang X., Yang F.-Z., Ren B. (2015). Rational fabrication
of a gold-coated AFM TERS tip by pulsed electrodeposition. Nanoscale.

[128] Huang T.-X., Li C.-W., Yang L.-K., Zhu J.-F., Yao X., Liu C., Lin K.-Q., Zeng Z.-C., Wu S.-S., Wang X. (2018). Rational fabrication of silver-coated AFM TERS tips
with a high enhancement and long lifetime. Nanoscale.

[129] Williams C., Roy D. (2008). Fabrication of gold
tips suitable
for tip-enhanced Raman spectroscopy. J. Vac.
Sci. Technol. B.

[130] Neacsu C. C., Berweger S., Raschke M. B. (2007). Tip-enhanced Raman
imaging and nanospectroscopy: sensitivity, symmetry, and selection
rules. Nanobiotechnology.

[131] Lloyd J. S., Williams A., Rickman R. H., McCowen A., Dunstan P. R. (2011). Reproducible electrochemical etching
of silver probes
with a radius of curvature of 20 nm for tip-enhanced Raman applications. Appl. Phys. Lett..

[132] Zhang W., Yeo B. S., Schmid T., Zenobi R. (2007). Single Molecule
Tip-Enhanced Raman Spectroscopy with Silver Tips. J. Phys. Chem. C.

[133] Ren B., Picardi G., Pettinger B. (2004). Preparation of Gold Tips Suitable
for Tip-Enhanced Raman Spectroscopy and Light Emission by Electrochemical
Etching. Rev. Sci. Instrum..

[134] Dickmann K., Demming F., Jersch J. (1996). New etching
procedure
for silver scanning tunneling microscopy tips. Rev. Sci. Instrum..

[135] Iwami M., Uehara Y., Ushioda S. (1998). Preparation of silver
tips for scanning tunneling microscopy imaging. Rev. Sci. Instrum..

[136] Stadler J., Schmid T., Zenobi R. (2010). Nanoscale Chemical
Imaging Using Top-Illumination Tip-Enhanced Raman Spectroscopy. Nano Lett..

[137] Bryant P. J., Kim H. S., Zheng Y. C., Yang R. (1987). Technique
for shaping scanning tunneling microscope tips. Rev. Sci. Instrum..

[138] Melmed A. J. (1991). The art
and science and other aspects of making sharp
tips. J. Vac. Sci. Technol. B.

[139] Opilik L., Dogan Ü., Szczerbiński J., Zenobi R. (2015). Degradation of silver near-field optical probes and
its electrochemical reversal. Appl. Phys. Lett..

[140] Wang X., Liu Z., Zhuang M., Zhang H., Wang X., Xie Z., Wu D., Ren B., Tian Z. (2007). Tip-enhanced Raman spectroscopy for investigating adsorbed
species
on a single-crystal surface using electrochemically prepared Au tips. Appl. Phys. Lett..

[141] Vasconcelos T. L., Archanjo B. S., Fragneaud B., Oliveira B. S., Riikonen J., Li C., Ribeiro D. S., Rabelo C., Rodrigues W. N., Jorio A. (2015). Tuning
Localized Surface Plasmon Resonance in Scanning Near-Field Optical
Microscopy Probes. ACS Nano.

[142] Vasconcelos T. L., Archanjo B. S., Oliveira B. S., Valaski R., Cordeiro R. C., Medeiros H. G., Rabelo C., Ribeiro A., Ercius P., Achete C. A., Jorio A., Cancado L. G. (2018). Plasmon-Tunable
Tip Pyramids: Monopole Nanoantennas for Near-Field Scanning Optical
Microscopy. Advanced Optical Materials.

[143] Kim S., Yu N., Ma X., Zhu Y., Liu Q., Liu M., Yan R. (2019). High external-efficiency
nanofocusing for lens-free
near-field optical nanoscopy. Nat. Photonics.

[144] Kumar N., Spencer S. J., Imbraguglio D., Rossi A. M., Wain A. J., Weckhuysen B. M., Roy D. (2016). Extending the plasmonic lifetime of tip-enhanced Raman spectroscopy
probes. Phys. Chem. Chem. Phys..

[145] Martina I., Wiesinger R., Schreiner M. (2013). Micro-Raman
investigations of early stage silver corrosion products occurring
in sulfur containing atmospheres. J. Raman Spectrosc..

[146] Minceva-Sukarova B., Najdoski M., Grozdanov I., Chunnilall C. J. (1997). Raman spectra of thin solid films of some metal sulfides. J. Mol. Struct..

[147] Szczerbiński J., Yin H., Zhang Y.-J., Zhang F.-L., Li J.-F., Zenobi R. (2020). Preserving
Plasmonic Nanostructures
from Laser-Induced Deactivation by a Protective Dielectric Shell. J. Phys. Chem. C.

[148] Opilik L., Dogan U. z., Li C.-Y., Stephanidis B., Li J.-F., Zenobi R. (2016). Chemical production of thin protective
coatings on optical nanotips for tip-enhanced Raman spectroscopy. J. Phys. Chem. C.

[149] Barrios C. A., Malkovskiy A. V., Kisliuk A. M., Sokolov A. P., Foster M. D. (2009). Highly Stable, Protected Plasmonic Nanostructures for
Tip Enhanced Raman Spectroscopy. J. Phys. Chem.
C.

[150] Taber B. N., Neill M. L., Thom T. N., Clapp O. D., Apkarian V. A., Lee J. (2023). In situ plasmonic tip
preparation
and validation techniques for scanning tunneling microscopy. Journal of Vacuum Science & Technology A.

[151] Mahapatra S., Li L., Schultz J. F., Jiang N. (2021). Methods to
fabricate and recycle plasmonic probes for ultrahigh vacuum scanning
tunneling microscopy-based tip-enhanced Raman spectroscopy. J. Raman Spectrosc..

[152] Zeng Z. C., Huang S. C., Wu D. Y., Meng L. Y., Li M. H., Huang T. X., Zhong J. H., Wang X., Yang Z. L., Ren B. (2015). Electrochemical Tip-Enhanced Raman
Spectroscopy. J. Am. Chem. Soc..

[153] Scherger J. D., Foster M. D. (2017). Tunable, liquid
resistant tip-enhanced
Raman spectroscopy probes: Toward label-free nano-resolved imaging
of biological systems. Langmuir.

[154] Pourbaix, M. Atlas of Electrochemical Equilibria in Aqueous Solutions; National Association of Corrosion Engineers: Houston, 1974.

[155] Vaidya P. D., Lopez-Sanchez J. A. (2017). Review of hydrogen production by
catalytic aqueous-phase reforming. ChemistrySelect.

[156] Navarro R. M., Pena M., Fierro J. (2007). Hydrogen production
reactions from carbon feedstocks: fossil fuels and biomass. Chem. Rev..

[157] Sabanes N. M., Driessen L. M. A., Domke K. F. (2016). Versatile Side-Illumination
Geometry for Tip-Enhanced Raman Spectroscopy at Solid/Liquid Interfaces. Anal. Chem..

[158] Yokota Y., Hayazawa N., Yang B., Kazuma E., Catalan F. C. I., Kim Y. (2019). Systematic Assessment of Benzenethiol
Self-Assembled Monolayers on Au(111) as a Standard Sample for Electrochemical
Tip-Enhanced Raman Spectroscopy. J. Phys. Chem.
C.

[159] Chen X., Brasiliense V., Van Duyne R. P. (2018). Operando
Observation of Molecular-Scale Manipulation Using Electrochemical
Tip-Enhanced Raman Spectroscopy. J. Phys. Chem.
C.

[160] Chen Z., Jiang S., Kang G., Nguyen D., Schatz G. C., Van Duyne R. P. (2019). Operando
Characterization of Iron
Phthalocyanine Deactivation during Oxygen Reduction Reaction Using
Electrochemical Tip-Enhanced Raman Spectroscopy. J. Am. Chem. Soc..

[161] Huang S.-C., Wang X., Zhao Q.-Q., Zhu J.-F., Li C.-W., He Y.-H., Hu S., Sartin M. M., Yan S., Ren B. (2020). Probing nanoscale spatial distribution of plasmonically
excited hot carriers. Nat. Commun..

[162] Huang S.-C., Zhao Q.-Q., Feng H.-S., Ma H., Zhao L.-b., Wang X., Ren B. (2023). Probing the Intermediate
in the Electrochemical Reduction of Nitrobenzene Derivative by EC-TERS. J. Phys. Chem. C.

[163] Sabanes N. M., Ohto T., Andrienko D., Nagata Y., Domke K. F. (2017). Electrochemical TERS Elucidates Potential-Induced
Molecular Reorientation of Adenine/Au(111). Angew. Chem., Int. Ed..

[164] Pfisterer J. H. K., Baghernejad M., Giuzio G., Domke K. F. (2019). Reactivity
mapping of nanoscale defect chemistry under electrochemical reaction
conditions. Nat. Commun..

[165] Bao Y.-F., Cao M.-F., Wu S.-S., Huang T.-X., Zeng Z., Li M.-H., Wang X., Ren B. (2020). Atomic Force
Microscopy-Based Top-Illumination Electrochemical Tip-Enhanced Raman
Spectroscopy. Anal. Chem..

[166] Domke K. F., Pettinger B. (2009). In situ discrimination
between axially
complexed and ligand-free Co porphyrin on Au(111) with tip-enhanced
Raman spectroscopy. ChemPhysChem.

[167] Haynes C. L., Schatz G. C., Weiss P. S. (2020). Virtual
Issue in
Honor of Prof. Richard Van Duyne (1945–2019). Anal. Chem..

[168] Nguyen D., Kang G., Chiang N., Chen X., Seideman T., Hersam M. C., Schatz G. C., Van Duyne R. P. (2018). Probing
molecular-scale catalytic interactions between oxygen and cobalt phthalocyanine
using tip-enhanced Raman spectroscopy. J. Am.
Chem. Soc..

[169] Su H.-S., Feng H.-S., Zhao Q.-Q., Zhang X.-G., Sun J.-J., He Y., Huang S.-C., Huang T.-X., Zhong J.-H., Wu D.-Y., Ren B. (2020). Probing the Local Generation
and Diffusion of Active Oxygen Species on a Pd/Au Bimetallic Surface
by Tip-Enhanced Raman Spectroscopy. J. Am. Chem.
Soc..

[170] Zhong J. H., Jin X., Meng L. Y., Wang X., Su H. S., Yang Z. L., Williams C. T., Ren B. (2017). Probing the
electronic and catalytic properties of a bimetallic surface with 3
nm resolution. Nat. Nanotechnol..

[171] Su H. S., Zhang X. G., Sun J. J., Jin X., Wu D. Y., Lian X. B., Zhong J. H., Ren B. (2018). Real-Space
Observation of Atomic Site-Specific Electronic Properties of a Pt
Nanoisland/Au(111) Bimetallic Surface by Tip-Enhanced Raman Spectroscopy. Angew. Chem., Int. Ed..

[172] Filez M., Walke P., Le-The H., Toyouchi S., Peeters W., Tomkins P., Eijkel J. C. T., De
Feyter S., Detavernier C., De Vos D. E., Uji-I H., Roeffaers M. B. J. (2024). Nanoscale Chemical Diversity of Coke Deposits on Nanoprinted
Metal Catalysts Visualized by Tip-Enhanced Raman Spectroscopy. Adv. Mater..

[173] Vermeiren W., Gilson J.-P. (2009). Impact of zeolites on the petroleum
and petrochemical industry. Top. Catal..

[174] Vogt E. T. C., Weckhuysen B. M. (2015). Fluid catalytic
cracking: recent
developments on the grand old lady of zeolite catalysis. Chem. Soc. Rev..

[175] Buurmans I. L. C., Ruiz-Martínez J., Knowles W. V., van der
Beek D., Bergwerff J. A., Vogt E. T. C., Weckhuysen B. M. (2011). Catalytic
activity in individual cracking catalyst particles imaged throughout
different life stages by selective staining. Nat. Chem..

[176] Buurmans I. L. C., Ruiz-Martínez J., van Leeuwen S. L., van der Beek D., Bergwerff J. A., Knowles W. V., Vogt E. T. C., Weckhuysen B. M. (2012). Staining of Fluid-Catalytic-Cracking
Catalysts: Localising Brønsted Acidity within a Single Catalyst
Particle. Chem.—Eur. J..

[177] Sprung C., Weckhuysen B. M. (2014). Dispersion
and Orientation of Zeolite
ZSM-5 Crystallites within a Fluid Catalytic Cracking Catalyst Particle. Chem.—Eur. J..

[178] Whiting G. T., Meirer F., Valencia D., Mertens M. M., Bons A.-J., Weiss B. M., Stevens P. A., De Smit E., Weckhuysen B. M. (2014). Selective staining of Brønsted acidity in zeolite
ZSM-5-based catalyst extrudates using thiophene as a probe. Phys. Chem. Chem. Phys..

[179] Cai Z.-F., Tang Z.-X., Zhang Y., Kumar N. (2024). Mechanistic
Understanding of Oxygen Activation on Bulk Au(111) Surface Using Tip-Enhanced
Raman Spectroscopy. Angew. Chem., Int. Ed..

[180] Huang Y.-F., Zhu H.-P., Liu G.-K., Wu D.-Y., Ren B., Tian Z.-Q. (2010). When the signal is not from the original molecule to
be detected: chemical transformation of para-aminothiophenol on Ag
during the SERS measurement. J. Am. Chem. Soc..

[181] Zhao L. B., Zhang M., Huang Y. F., Williams C. T., Wu D. Y., Ren B., Tian Z. Q. (2014). Theoretical
study
of plasmon-enhanced surface catalytic coupling reactions of aromatic
amines and nitro compounds. J. Phys. Chem. Lett..

[182] van Schrojenstein Lantman E. M., Deckert-Gaudig T., Mank A. J., Deckert V., Weckhuysen B. M. (2012). Catalytic
processes monitored at the nanoscale with tip-enhanced Raman spectroscopy. Nat. Nanotechnol..

[183] Bhattarai A., El-Khoury P. Z. (2019). Nanoscale Chemical Reaction Imaging
at the Solid-Liquid Interface via TERS. J. Phys.
Chem. Lett..

[184] Wang C.-F., O’Callahan B.
T., Kurouski D., Krayev A., El-Khoury P. Z. (2020). The Prevalence of Anions at Plasmonic
Nanojunctions: A Closer Look at p-Nitrothiophenol. J. Phys. Chem. Lett..

[185] Sun M. T., Zhang Z. L., Zheng H. R., Xu H. X. (2012). In-situ
plasmon-driven chemical reactions revealed by high vacuum tip-enhanced
Raman spectroscopy. Sci. Rep..

[186] Zhang Z. L., Chen L., Sun M. T., Ruan P. P., Zheng H. R., Xu H. X. (2013). Insights into the
nature of plasmon-driven
catalytic reactions revealed by HV-TERS. Nanoscale.

[187] Wang R., Li J., Rigor J., Large N., El-Khoury P. Z., Rogachev A. Y., Kurouski D. (2020). Direct Experimental
Evidence of Hot Carrier-Driven Chemical Processes in Tip-Enhanced
Raman Spectroscopy (TERS). J. Phys. Chem. C.

[188] Li Z., Kurouski D. (2021). Tip-Enhanced Raman
Analysis of Plasmonic and Photocatalytic
Properties of Copper Nanomaterials. J. Phys.
Chem. Lett..

[189] Patil S. J., Kurouski D. (2024). Nanoscale Imaging of Palladium-Enhanced
Photocatalytic Reduction of 4-Nitrothiophenol on Tungsten Disulfide
Nanoplates. Nano Lett..

[190] Li Z. D., Wang R., Kurouski D. (2020). Nanoscale
Photocatalytic
Activity of Gold and Gold-Palladium Nanostructures Revealed by Tip-Enhanced
Raman Spectroscopy. J. Phys. Chem. Lett..

[191] Sytwu K., Vadai M., Dionne J. A. (2019). Bimetallic
nanostructures:
combining plasmonic and catalytic metals for photocatalysis. Advances in Physics: X.

[192] Li Z. D., Kurouski D. (2020). Elucidation of Photocatalytic Properties
of Gold-Platinum Bimetallic Nanoplates Using Tip-Enhanced Raman Spectroscopy. J. Phys. Chem. C.

[193] Li Z., Kurouski D. (2021). Probing the Redox Selectivity on
Au@Pd and Au@Pt Bimetallic
Nanoplates by Tip-Enhanced Raman Spectroscopy. ACS Photonics.

[194] Li Z., Kurouski D. (2021). Probing the
plasmon-driven Suzuki–Miyaura coupling
reactions with cargo-TERS towards tailored catalysis. Nanoscale.

[195] Li H., Tang Z.-X., Zhang J.-X., Zhang X.-B., Zhang Y.-F., Zhang Y., Zhang Y., Dong Z.-C. (2023). Probing coverage-dependent
adsorption configuration and on-surface dimerization by single-molecule
tip-enhanced Raman spectroscopy. Appl. Phys.
A: Mater. Sci. Process..

[196] Sun J.-J., Su H.-S., Yue H.-L., Huang S.-C., Huang T.-X., Hu S., Sartin M. M., Cheng J., Ren B. (2019). Role of adsorption
orientation in surface plasmon-driven coupling
reactions studied by tip-enhanced Raman spectroscopy. journal of physical chemistry letters.

[197] Sun M., Zhang Z., Kim Z. H., Zheng H., Xu H. (2013). Plasmonic
scissors for molecular design. Eur. J. Chem..

[198] Zhou L., Zhang C., McClain M. J., Manjavacas A., Krauter C. M., Tian S., Berg F., Everitt H. O., Carter E. A., Nordlander P., Halas N. J. (2016). Aluminum nanocrystals
as a plasmonic photocatalyst for hydrogen dissociation. Nano Lett..

[199] Mukherjee S., Zhou L., Goodman A. M., Large N., Ayala-Orozco C., Zhang Y., Nordlander P., Halas N. J. (2014). Hot-electron-induced dissociation of H2 on gold nanoparticles
supported on SiO2. J. Am. Chem. Soc..

[200] Szczerbiński J., Gyr L., Kaeslin J., Zenobi R. (2018). Plasmon-driven
photocatalysis leads to products known from E-beam and X-ray-induced
surface chemistry. Nano Lett..

[201] Szczerbiński J., Metternich J. B., Goubert G., Zenobi R. (2020). How peptides
dissociate in plasmonic hot spots. Small.

[202] Wang R.-P., Yang B., Fu Q., Zhang Y., Zhu R., Dong X.-R., Zhang Y., Wang B., Yang J.-L., Luo Y. (2021). Raman
Detection of Bond Breaking and Making of a Chemisorbed
Up-Standing Single Molecule at Single-Bond Level. J. Phys. Chem. Lett..

[203] Gelder E. A., Jackson S. D., Lok C. M. (2005). The hydrogenation
of nitrobenzene to aniline: a new mechanism. Chem. Commun..

[204] Cai Z.-F., Manae M. A., Tang Z.-X., Moskalenko A., Zhang Y., Richardson J. O., Kumar N. (2025). Mechanistic Insights
into Nitroarene Hydrogenation Dynamics on Pt(111) via In Situ Tip-Enhanced
Raman Spectroscopy. J. Am. Chem. Soc..

[205] de Ruiter J., An H., Wu L., Gijsberg Z., Yang S., Hartman T., Weckhuysen B. M., van der Stam W. (2022). Probing the Dynamics of Low-Overpotential CO2-to-CO
Activation on Copper Electrodes with Time-Resolved Raman Spectroscopy. J. Am. Chem. Soc..

[206] Osawa M. (1997). Dynamic Processes in Electrochemical Reactions Studied by Surface-Enhanced
Infrared Absorption Spectroscopy (SEIRAS). Bull.
Chem. Soc. Jpn..

[207] Polcari D., Dauphin-Ducharme P., Mauzeroll J. (2016). Scanning Electrochemical
Microscopy: A Comprehensive Review of Experimental Parameters from
1989 to 2015. Chem. Rev..

[208] Kurouski D., Mattei M., Van Duyne R. P. (2015). Probing
Redox Reactions at the Nanoscale with Electrochemical Tip-Enhanced
Raman Spectroscopy. Nano Lett..

[209] Touzalin T., Joiret S., Lucas I. T., Maisonhaute E. (2019). Electrochemical
tip-enhanced Raman spectroscopy imaging with 8 nm lateral resolution. Electrochem. commun..

[210] Mattei M., Kang G., Goubert G., Chulhai D. V., Schatz G. C., Jensen L., Van Duyne R. P. (2017). Tip-Enhanced
Raman Voltammetry: Coverage Dependence and Quantitative Modeling. Nano Lett..

[211] Kang G., Yang M. W., Mattei M. S., Schatz G. C., Van Duyne R. P. (2019). In Situ Nanoscale Redox Mapping Using Tip-Enhanced
Raman Spectroscopy. Nano Lett..

[212] Fiocco A., Pavlic A. A., Kanoufi F., Maisonhaute E., Noël J.-M., Lucas I. T. (2024). Electrochemical
Tip-Enhanced Raman
Spectroscopy for the Elucidation of Complex Electrochemical Reactions. Anal. Chem..

[213] Huang T.-X., Cong X., Wu S.-S., Wu J.-B., Bao Y.-F., Cao M.-F., Wu L., Lin M.-L., Wang X., Tan P.-H. (2024). Visualizing the structural
evolution of individual active sites in MoS2 during electrocatalytic
hydrogen evolution reaction. Nature Catalysis.

[214] Umakoshi T., Fukuda S., Iino R., Uchihashi T., Ando T. (2020). High-speed near-field fluorescence
microscopy combined with high-speed
atomic force microscopy for biological studies. Biochimica et Biophysica Acta (BBA) - General Subjects.

[215] Kato R., Moriyama T., Umakoshi T., Yano T.-a., Verma P. (2022). Ultrastable tip-enhanced hyperspectral
optical nanoimaging for defect
analysis of large-sized WS2 layers. Science
Advances.

